# Multifunctional Ionic Liquid Enabling Cation Homogenization and Lead Chelation for Pure‐Iodide Wide‐Bandgap Perovskite Solar Cells

**DOI:** 10.1002/advs.77993

**Published:** 2026-09-27

**Authors:** Kai Wu, Lei Yang, Guoqing Du, Xin Wang, Jiali Wei, Tiantian Li, Pengfei Zhang, Xiaodan Zhang, Fuhua Hou

**Affiliations:** ^1^ Key Laboratory of Semiconductor Photovoltaic Technology and Energy Materials of Inner Mongolia Autonomous Region School of Physical Science and Technology Inner Mongolia University Hohhot China; ^2^ Institute of Photoelectronic Thin Film Devices and Technology Tianjin Key Laboratory of Efficient Utilization of Solar Energy State Key Laboratory of Photovoltaic Materials and Cells Nankai University Tianjin China; ^3^ Institutes of Biomedical Sciences School of Life Sciences Inner Mongolia University Hohhot China; ^4^ Research Center for Quantum Physics and Technologies Inner Mongolia University Hohhot China

**Keywords:** A‐site cation homogenization, crystallization kinetics, lead chelation, multifunctional ionic liquid, pure‐iodide wide‐bandgap perovskite solar cells

## Abstract

Wide‐bandgap perovskite solar cells (WBG‐PSCs) are essential for high‐efficiency tandem photovoltaics, yet conventional mixed‐halide formulations suffer from photoinduced halide segregation. While pure‐iodide WBG‐PSCs offer a fundamental solution, their development is hindered by the distinct crystallization kinetics of A‐site cations, which lead to severe compositional inhomogeneity, coupled with lead toxicity. Herein, we report a multifunctional ionic liquid, 1‐propylsulfonate‐3‐methylimidazolium trifluoromethanesulfonate ([SO_3_H‐PMIm][OTf]), that addresses these challenges. The additive interacts with CsI through sulfonate‐Cs^+^ coordination bonds to increase CsI solubility, anchors methylammonium (MA) and dimethylammonium (DMA) cations via hydrogen bonding, and coordinates with undercoordinated Pb^2+^ ions through the ─SO_3_^−^. These synergistic interactions suppress colloidal aggregation in the precursor solution, while this coordination effect lowers the crystallization temperature of Cs‐based perovskites, enabling synchronous crystallization with their organic‐based counterparts. Consequently, homogeneous A‐site cation distribution across both in‐plane and out‐of‐plane dimensions is achieved. The resulting Cs_0.3_DMA_0.2_MA_0.5_PbI_3_ pure‐iodide WBG‐PSCs deliver a champion efficiency of 22.02% with an open‐circuit voltage (*V*_OC_) of 1.231 V and retain 90% of initial efficiency after 3000 h under the ISOS‐D‐1 protocol. Pb^2+^ chelation and hydrophobic ─CF_3_ mitigate lead leakage by over 58%. This strategy provides a versatile strategy for overcoming cation inhomogeneity and lead toxicity, enabling efficient, stable, and safe pure‐iodide perovskite photovoltaics.

## Graphical abstract

This work presents a multifunctional ionic liquid, [SO_3_H‐PMIm][OTf], that addresses A‐site cation inhomogeneity and lead toxicity in pure‐iodide wide‐bandgap perovskite solar cells. The additive enables homogeneous cation distribution, achieving 22.02% efficiency with 90% retention after 3000 h and reducing lead leakage by over 58%, thereby comprehensively enhancing device efficiency, stability, and safety.

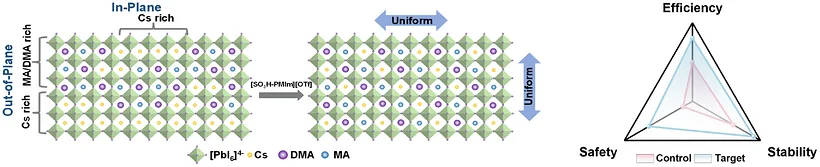

## 1 Introduction

Wide‐bandgap perovskite solar cells (WBG‐PSCs) (energy gap (*E*_g_) > 1.65 eV), as a critical component of perovskite‐based tandem photovoltaics, offer a feasible route to surpass the Shockley–Queisser (S–Q) limit of single‐junction devices [1, 2, 3]. The fabrication of conventional WBG‐PSCs is primarily achieved by substituting iodide with bromide, wherein the bandgap is tuned to exceed 1.65 eV by adjusting the bromide content to meet the requirements of tandem devices. However, this mixed‐halide strategy suffers from severe photoinduced phase segregation when the bromide content exceeds 20%, forming iodide‐rich domains that act as carrier traps, exacerbating non‐radiative recombination and causing open‐circuit voltage (*V*_OC_) loss and device failure [4, 5, 6]. Essentially, halide segregation originates from the migration of halide ions along vacancy defects, and halogen vacancies, owing to their low formation energy, represent one of the most prevalent intrinsic defects in perovskites [7]. Although researchers have attempted to suppress phase segregation through crystallization regulation [8, 9], defect passivation [10, 11], and bromide content reduction [12, 13], the risk of halide segregation remains challenging to fundamentally eliminate for the relatively high bromide concentrations required in tandem applications (∼15% for silicon‐based tandems and ∼30% for all‐perovskite tandems). Consequently, developing single‐halide‐based wide‐bandgap perovskites is considered a fundamental approach to definitively resolve photo‐induced halide segregation.

Pure‐iodide WBG‐PSCs can be realized via A‐site cation engineering. By introducing larger cations, such as methylammonium (MA), formamidinium (FA), or dimethylammonium (DMA), into the A‐site, cesium (Cs)‐rich pure‐iodide WBG‐PSCs with a desirable tolerance factor and robust phase stability can be constructed (e.g., Cs_0.5_FA_0.5_PbI_3_ and Cs_0.3_DMA_0.2_MA_0.5_PbI_3_) [14, 15, 16]. However, the introduction of multiple A‐site cations introduces new challenges, as the pronounced disparities in their crystallization behaviors hinder synchronous nucleation and growth, ultimately leading to severe compositional inhomogeneity. The core issue underlying this inhomogeneity lies in the substantial difference in crystallization temperatures between Cs‐based and organic‐cation‐based perovskites. FA/MA/DMA‐based perovskites crystallize readily below 150°C, whereas the formation of the photoactive black phase of CsPbI_3_ typically requires temperatures exceeding 180°C (and up to 340°C) [17]. More critically, in organic solvents such as DMF and DMSO, CsPbI_3_ exhibits lower solubility and faster precipitation than its organic‐cation‐based counterparts with the same anion [18]. This dual disparity in solubility and kinetics leads to preferential nucleation and crystallization of Cs‐rich phases at the bottom interface during annealing of mixed‐cation films, while organic‐rich phases crystallize later at the surface, ultimately forming severe vertical compositional stratification along the film thickness direction. Another prominent consequence is the presence of impurity phases, primarily the residual non‐photoactive δ‐CsPbI_3_ phase. On the other hand, the existence of micron‐scale Cs^+^ aggregation in the precursor solution, which stems from the strong Coulombic interaction between Cs^+^ and the [PbI_x_]^n−^ framework that forms CsI‐PbI_2_ colloidal clusters, inevitably leads to significant in‐plane compositional inhomogeneity between Cs and organic cations in the resultant perovskite films [19]. In fact, the perovskite precursor solution is actually a colloid rather than a true solution. The size and concentration of such colloidal particles affect crystallization nucleation, while the large‐sized colloidal particles formed by aggregation alter the local effective concentration and nucleation behavior, ultimately giving rise to the observed in‐plane cation inhomogeneity in the resulting perovskite films. Consequently, this cation inhomogeneity across both in‐plane and out‐of‐plane dimensions induces energetic disorder and introduces localized non‐radiative recombination centers, ultimately severely impairing device efficiency and long‐term operational stability.

Achieving the synergistic and homogeneous assembly of Cs^+^ with organic cations into high‐quality perovskite phases necessitates kinetic regulation of the crystallization process. Notably, most reported approaches for pure‐iodide WBG perovskites have focused on directing the precipitation pathway toward beneficial intermediate phases, which subsequently transform into the desired perovskite structure. Examples include the use of intermediate phases such as Cs_2_PbI_2_Cl_2_ or Cs_4_PbI_6_ to mediate phase evolution and suppress the formation of secondary phases [14, 15, 20, 21]. Although these methods have achieved considerable success, they rely on the formation and subsequent conversion of specific intermediate phases, which increases process complexity and fails to fundamentally address the issues of cation diffusion kinetics during crystallization. More importantly, these strategies primarily focus on modulating the crystallization or annealing stages, with limited attention paid to the root cause of in‐plane cation inhomogeneity, namely colloidal aggregation and compositional non‐uniformity in the precursor solution stage. An alternative strategy involves directly lowering the crystallization energy barrier of Cs‐based perovskites through additives that coordinate with Cs^+^ [22], offering a more direct route for kinetic regulation. In contrast to indirect pathways relying on specific intermediates, modifies the chemical environment of CsI through sulfonate‐Cs^+^ interaction, which reduces the effective electrostatic attraction between Cs^+^ and I^−^, fundamentally narrowing the crystallization temperature disparity between Cs‐based and organic perovskites while simultaneously improving ion distribution uniformity in the precursor solution, holding promise for achieving synchronous crystallization of multiple cations. This approach of directly lowering the energy barrier remains relatively underexplored in pure‐iodide WBG‐PSCs, yet it holds significant potential for realizing simpler and more universal crystallization control. Among various additives employed in WBG‐PSCs, ionic liquids (ILs) have been widely adopted due to their advantages such as environmental friendliness, low toxicity, non‐volatility, high stability, and flexible multifunctional structural design. However, existing studies have primarily focused on utilizing the functional groups of ILs for defect passivation and crystallization regulation, while the issue of compositional inhomogeneity of cations has rarely been addressed [23, 24].

Previous studies have separately explored modification of CsI through sulfonate coordination in all‐inorganic CsPbI_3_ systems, cation homogenization via intermediate phase engineering, and ionic liquid additives for defect passivation. However, none of these approaches simultaneously tackle the root cause of cation inhomogeneity, colloidal aggregation in the precursor solution, nor do they integrate multiple functions into a single additive. This work, for the first time, combines sulfonate‐Cs^+^ coordination, A‐site cation homogenization, and lead chelation into one multifunctional ionic liquid, offering a comprehensive solution to the key challenges facing pure‐iodide WBG‐PSCs. Herein, we first propose an additive engineering strategy by incorporating a multifunctional IL, 1‐propylsulfonate‐3‐methylimidazolium trifluoromethanesulfonate ([SO_3_H‐PMIm][OTf]), to address the issue of cation inhomogeneity across both in‐plane and out‐of‐plane dimensions in Cs_0.3_DMA_0.2_MA_0.5_PbI_3_ pure‐iodide WBG perovskites. In the precursor solution, the sulfonate group of the cation coordinates with Cs^+^, modifying the chemical environment of CsI, which enhances its solubility. Simultaneously, the sulfonate anion interacts with Pb^2+^ and anchors organic cations via hydrogen bonding, concurrently increasing the negative surface charge of the colloids and thereby enhancing electrostatic repulsion between them. These synergistic effects collectively inhibit colloidal aggregation in the precursor solution and enhance its uniformity. During crystallization, this coordination between the sulfonate group and Cs^+^ lowers the crystallization energy barrier of Cs‐based perovskites, enabling the synchronous formation of Cs‐rich and organic‐rich phases at approximately 100°C, while retarding the overall crystallization rate to promote ordered grain growth, ultimately achieving a homogeneous distribution of Cs and organic cations both in‐plane and out‐of‐plane. As a result, the target pure‐iodide WBG‐PSCs delivered a champion power conversion efficiency of 22.02%. Furthermore, the ─CF_3_ group enhances hydrophobicity, which in turn improves the stability of the WBG‐PSCs, while the sulfonate moiety chelates Pb^2+^ to mitigate lead leakage. This integrated molecular design simultaneously addresses the efficiency, stability, and environmental safety concerns associated with Cs_0.3_DMA_0.2_MA_0.5_PbI_3_ pure‐iodide WBG‐PSCs.

## 2 Results and Discussion

**FIGURE 1:**
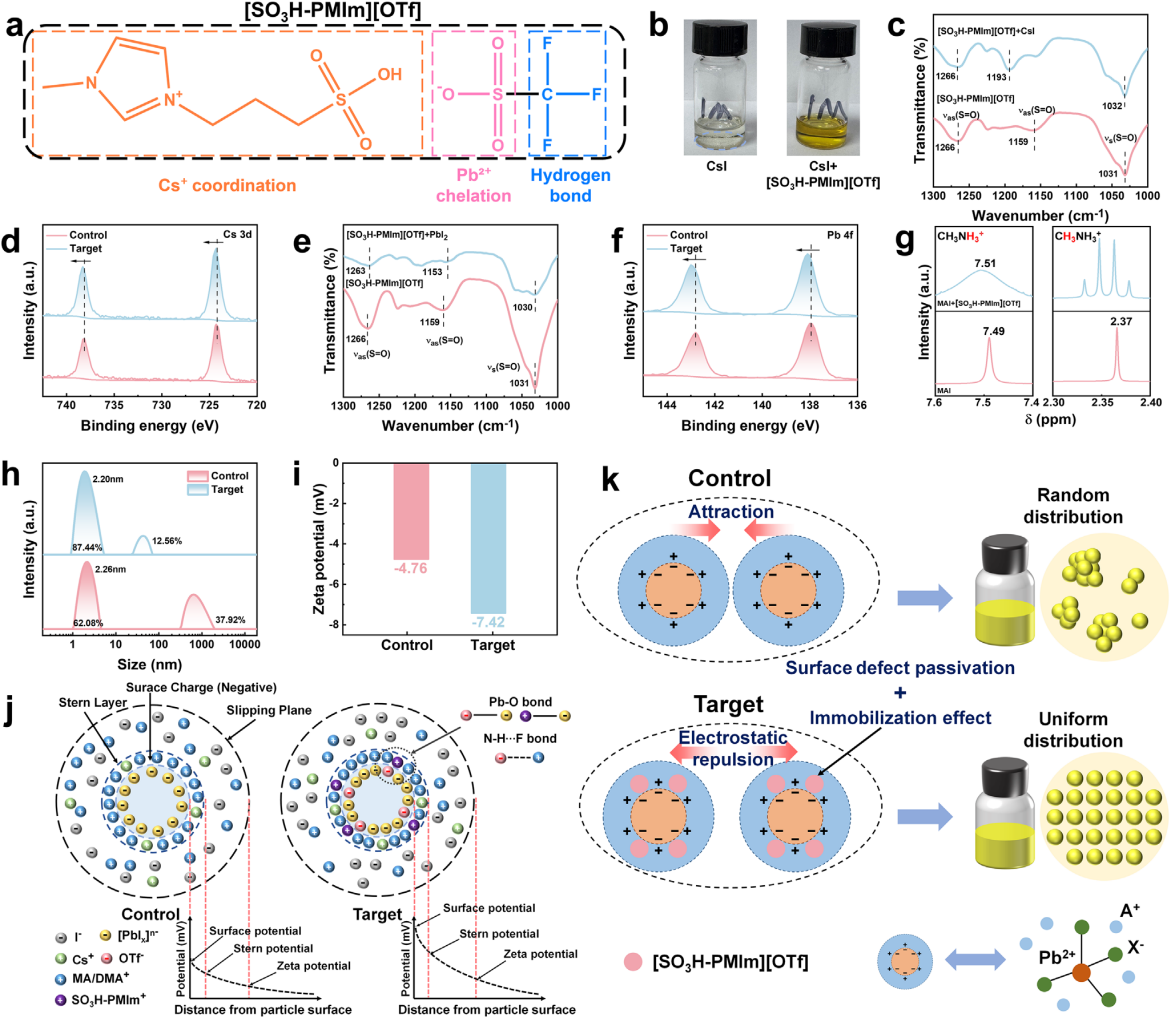
(a) Molecular structure of [SO_3_H‐PMIm][OTf] enabling hydrogen bonding, Pb‐chelating, and Cs^+^ coordination with perovskites. (b) 1 m CsI with or without [SO_3_H‐PMIm][OTf] dissolved in DMF: DMSO = 7:3. (c) FTIR spectra of [SO_3_H‐PMIm][OTf] and CsI + [SO_3_H‐PMIm][OTf]. (d) XPS spectra of Cs 3d. (e) FTIR spectra of [SO_3_H‐PMIm][OTf] and PbI_2_ + [SO_3_H‐PMIm][OTf]. (f) XPS spectra of Pb 4f. (g) ^1^H NMR spectra of MAI and MAI + [SO_3_H‐PMIm][OTf]. (h) DLS analysis of perovskite precursor solutions. (i) Zeta potential for the perovskite precursor solutions. (j) Schematic illustration of the surface charge and zeta potential difference between control and target colloidal inks. (k) Schematic illustration of the charge distribution and colloidal state of the perovskite colloidal electric double layer after the addition of [SO_3_H‐PMIm][OTf].

Since the origin of A‐site cation inhomogeneity can be traced back to the precursor solution stage, the formation of uniform perovskite films is intrinsically linked to the state of the perovskite precursor. In the precursor solution, perovskite components exist as colloids stabilized by an electrical double layer (EDL), which is formed through attractive interactions between negatively charged iodoplumbate ions and surrounding organic cations. Therefore, precise regulation of the solution chemistry is a prerequisite for obtaining homogeneous perovskite films [25, 26]. To address colloidal aggregation and inhomogeneity, we propose an additive strategy, as illustrated in Figure 1a, which functions by coordinating with Cs^+^ through its sulfonate groups on one hand and enhancing EDL stability by strengthening interactions between iodoplumbate ions and organic cations on the other. [SO_3_H‐PMIm][OTf] was selected for its triple functionality: sulfonate‐Cs^+^ coordination, chelating with lead ions, and forming hydrogen bonds with MA/DMA^+^ via its ─CF_3_ group. These interactions collectively hinder colloidal aggregation and improve solution uniformity.

Industrially, sulfonates are typical adsorbents for Cs^+^ capture in nuclear wastewater, suggesting that sulfonate‐based materials may coordinate with Cs^+^. Upon adding [SO_3_H‐PMIm][OTf] to CsI, the most pronounced change is the substantially increased solubility of CsI in DMF/DMSO solvents (Figure 1b), demonstrating a strong interaction between Cs^+^ and [SO_3_H‐PMIm][OTf]. Fourier‐transform infrared spectroscopy (FTIR) and X‐ray photoelectron spectroscopy (XPS) were employed to investigate this interaction. As shown in Figure 1c, pristine [SO_3_H‐PMIm][OTf] exhibits three characteristic FTIR peaks, with the bands at 1159 and 1031 cm^−1^ attributed to the asymmetric and symmetric stretching vibrations of S═O in the sulfonate group of the cation [22]. In the presence of CsI, these S═O peaks shift notably, indicating a strong interaction between CsI and the ─SO_3_^−^ group. In XPS, the Cs 3d signal of the control perovskite film shifts toward higher binding energy (Figure 1d), confirming the coordination between the sulfonate group and Cs^+^, reducing the effective electrostatic attraction between Cs^+^ and I^−^, which consequently lowers the crystallization temperature of CsPbI_3_.

Specifically, when CsPbI_3_ wet films are annealed at 90°C for 15 min, the sample incorporating [SO_3_H‐PMIm][OTf] successfully forms the γ‐CsPbI_3_ phase, whereas the additive‐free sample, due to insufficient crystallization temperature, yields the δ‐CsPbI_3_ phase, which further confirms the role of the additive in reducing the crystallization temperature of Cs‐based perovskites (Figure S1). Through this coordination with [SO_3_H‐PMIm][OTf], the chemical environment of CsI is modified, reducing the effective electrostatic attraction between Cs^+^ and I^−^. This facilitates the incorporation of I^−^ into the PbI_2_ framework to form corner‐sharing [PbI_6_]^4−^ octahedra, thereby promoting CsPbI_3_ crystallization (Figure S2). This mechanism substantially reduces the crystallization temperature of Cs‐based perovskites, bringing it into close alignment with that of MA/DMA‐based counterparts. Consequently, Cs‐based and MA/DMA‐based perovskites crystallize simultaneously, effectively suppressing Cs‐MA/DMA phase segregation.

The ─SO_3_^−^ group exhibits strong local Lewis basicity and a pronounced coordination capability with Pb^2+^ ions, thereby effectively suppressing the formation of Pb‐related defects. FTIR spectroscopy reveals strong coordination between ─SO_3_^−^ and Pb^2+^, which significantly weakens the S═O bond, resulting in distinct shifts of its characteristic S═O peaks (Figure 1e) [27]. XPS measurements confirm this interaction, with the Pb 4f and I 3d peaks shifting toward higher binding energy (Figure 1f and Figure S3a), indicating coordination between Pb^2+^ and the ─SO_3_^−^ group. UV–vis absorption spectroscopy was further employed to validate these interactions (Figure S3b). Upon mixing [SO_3_H‐PMIm][OTf] with PbI_2_, a pronounced red shift in the absorption onset is observed, suggesting coordination between ─SO_3_^−^ and Pb^2+^ [28]. The red shift of the absorption band in the mixed solution is attributed to the coordination bonds formed between the organic ligand in [SO_3_H‐PMIm][OTf] and metal ions, which affect the conjugated structure of the chromophore. Additionally, the PbI_2_ solution exhibits a color change to yellow, indicative of complex formation between [SO_3_H‐PMIm][OTf] and PbI_2_ [29]. These results collectively confirm the strong interaction between [SO_3_H‐PMIm][OTf] and lead iodide.

Among halide ions, fluoride (F^−^) stands out due to its highest electronegativity and smallest ionic radius, enabling it to form the strongest hydrogen bonds with perovskite components. In Figure S4, upon addition of [SO_3_H‐PMIm][OTf], the C–H vibrations and N–H stretching vibration peaks of MAI and DMAI shift simultaneously, indicating the formation of hydrogen bonds between fluorine atoms and the MAI/DMAI cations [29, 30]. ^1^H nuclear magnetic resonance (NMR) spectroscopy was employed to investigate the interactions between MAI and [SO_3_H‐PMIm][OTf] before and after mixing, as shown in Figure 1g and Figure S5a. Compared to pristine MAI, the ‐NH_3_^+^ peak of MAI broadens and shifts from 7.49 to 7.51 ppm upon addition of [SO_3_H‐PMIm][OTf], suggesting the presence of N─H···F interactions. Furthermore, MAI exhibits a single methyl (─CH_3_) peak at 2.37 ppm, which splits into four peaks after the addition of [SO_3_H‐PMIm][OTf]. The hydrogen bonds formed between the ─NH_3_^+^ species and the ─CF_3_ group disrupt the symmetric hydrogen configuration of the sp^3^‐hybridized ─NH_3_^+^ relative to the ─CH_3_ group, causing the singlet to transform into a quartet [31]. This N─H···F interaction anchors the MA cations and suppresses the generation of MA^+^ vacancies [28, 29, 32]. Similarly, ^1^H NMR analysis of [SO_3_H‐PMIm][OTf] and DMAI reveals the same interaction mechanism as described above (Figure S5b,c). To further confirm the formation of hydrogen bonds within the perovskite, XPS analysis was conducted (Figure S5d). Upon incorporation of [SO_3_H‐PMIm][OTf], the N 1s peak of the perovskite film shifts toward higher binding energy, attributed to the reduced electron cloud density around nitrogen atoms resulting from N─H···F hydrogen bond formation, thereby confirming the hydrogen bonding interaction between the ILs and perovskite components.

Dynamic light scattering (DLS) was employed to investigate the effect of the additive on perovskite colloids. The control solution shows severe colloidal aggregation, with large colloids reaching ∼1000 nm (Figure 1h). This change likely originates from surface defects, primarily undercoordinated Pb^2+^ sites, on perovskite colloidal particles, which reduce surface charge density and weaken electrostatic repulsion between colloids, thereby destabilizing the colloidal system. The resulting disorderly motion and collision of colloids in solution promote non‐uniform aggregation and significant size variations. The incorporation of [SO_3_H‐PMIm][OTf] effectively prevents the formation of large colloids, ensuring that the host colloids remain predominantly distributed within a uniform, small‐size range. The zeta potential of the solution becomes more negative, changing from −4.76 to −7.42 mV (Figure 1i), reflecting the potential at the slipping plane of the EDL around perovskite colloids, which is a critical indicator of colloidal stability. [SO_3_H‐PMIm][OTf] coordinates with undercoordinated Pb^2+^ sites on the colloidal surface via ─SO_3_^−^ groups, thereby passivating positively charged defects and increasing both the negative charge density on the colloid surface and the charge density within the Stern layer, resulting in a larger absolute zeta potential. Notably, [SO_3_H‐PMIm]^+^ cations can also enter the Stern layer. Although positively charged and able to partially neutralize the negative colloid surface, their lower charge density and greater steric hindrance make them far less efficient in compensating surface charge than smaller cations like MA^+^, DMA^+^ and Cs^+^. Consequently, compared with a Stern layer occupied solely by smaller cations, the presence of [SO_3_H‐PMIm]^+^ dilutes the local positive charge density, further contributing to a more negative zeta potential. Additionally, the ─CF_3_ group of OTf^−^ forms hydrogen bonds with organic cations, anchoring organic cations in the vicinity of lead‐iodide frameworks and preventing colloidal aggregation induced by organic cations desorption (Figure 1j) [33].

In brief, the cation coordinates with Cs^+^ through its sulfonate group, increasing the solubility of CsI, while the incorporation of the anion effectively immobilizes the cations surrounding iodoplumbate ions through strong anchoring interactions. Simultaneously, it passivates defects on the surface of perovskite colloids and increases their negative surface charge, thereby enhancing electrostatic repulsion between colloids, effectively preventing aggregation, and ultimately stabilizing the colloidal system (Figure 1k). Consequently, the addition of [SO_3_H‐PMIm][OTf] stabilizes the EDL, thereby reducing colloidal aggregation and improving size uniformity, providing a prerequisite for the homogeneous distribution of A‐site cations [19].

**FIGURE 2:**
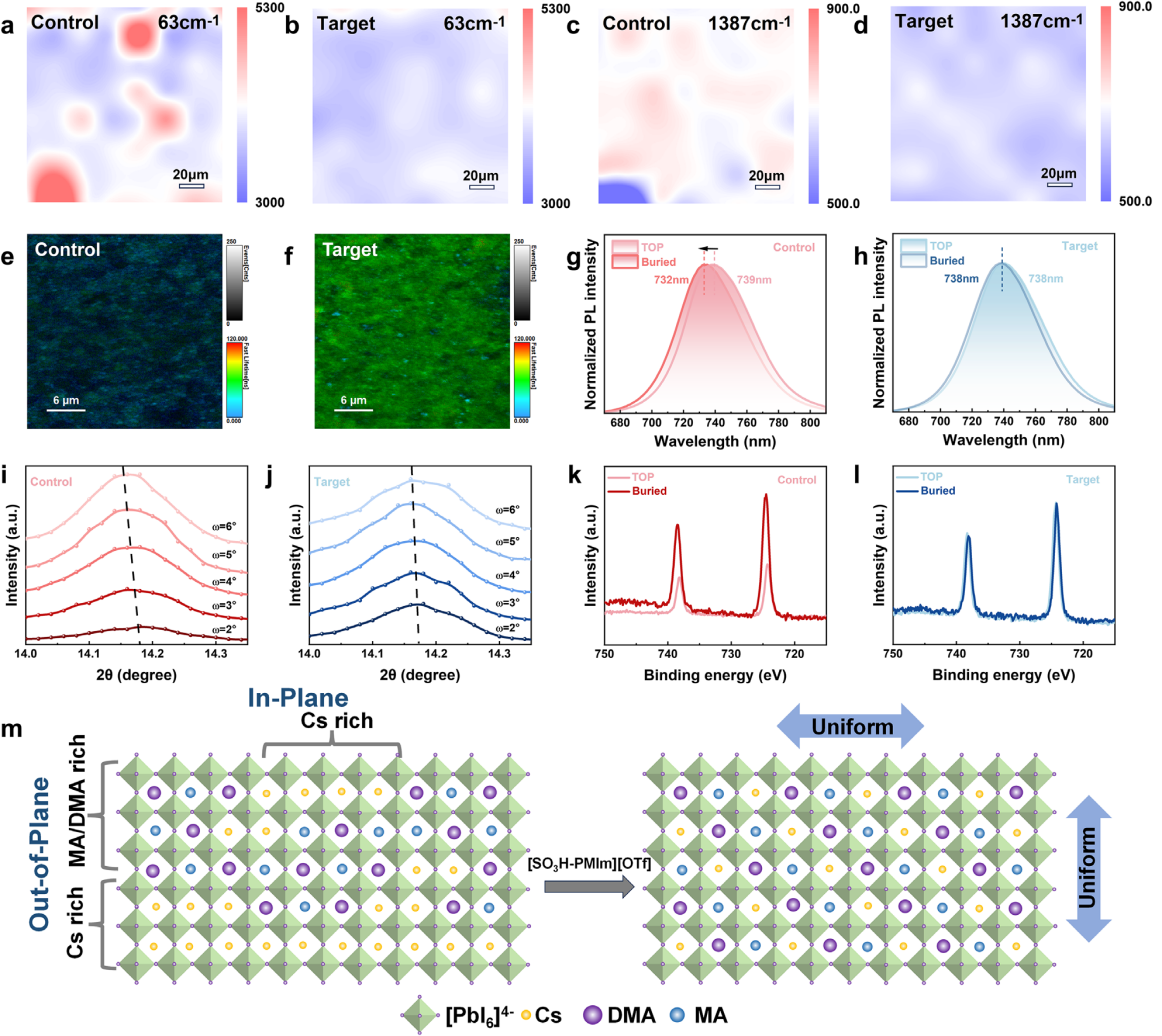
Raman mapping of (a) control and (b) target perovskite films at 63 cm^−1^. Raman mapping of (c) control and (d) target perovskite films at 1387 cm^−1^. PL intensity and lifetime mapping images of (e) control and (f) target perovskite films deposited on ITO substrates. PL from top and buried surface of (g) control and (h) target films. GIXRD from the top surface of the (i) control and (j) target perovskite films. XPS Cs 3d spectra of the top surface and buried interface of (k) control and (l) target perovskite films. (m) Schematic illustration of cation distribution.

Raman spectroscopy of the perovskite film was employed to investigate the distribution of Cs and MA/DMA. The Raman shift at 63 cm^−1^ corresponds to Cs–I vibrations, while the shift at 1387 cm^−1^ is attributed to the wagging vibrations of ─NH_2_ in MA/DMA (Figure 2a–d) [17, 22, 34, 35]. The Raman mapping reveals that the control pure‐iodide perovskite film exhibits an inhomogeneous Cs distribution (Figure 2a), which is further corroborated by the MA/DMA mapping, where MA/DMA‐rich regions correlate with Cs‐poor areas (Figure 2c). In contrast, the perovskite incorporating [SO_3_H‐PMIm][OTf] displays a substantially more uniform distribution of both Cs and MA/DMA (Figure 2b,d).

We further employed fluorescence lifetime imaging microscopy (FLIM) to investigate the in‐plane uniformity of the perovskite films. As shown in Figure 2e,f, the upper surface of the control film exhibits pronounced variations in both photoluminescence intensity and carrier lifetime, characterized by a random distribution pattern. This reflects compositional inhomogeneity within the plane of the film, with localized regions of Cs enrichment and depletion. In contrast, the target film displays nearly uniform intensity and lifetime distributions across the entire probed area in the FLIM images, indicating substantially improved homogeneity. Furthermore, the target film exhibits overall higher emission intensity and longer carrier lifetimes [36].

To further investigate charge recombination behavior and carrier lifetimes, steady‐state photoluminescence (PL) and time‐resolved photoluminescence (TRPL) measurements were conducted. The target film demonstrates higher PL intensity and prolonged carrier lifetimes (Figure S6a,b and Table S1), suggesting reduced trap density and consequently suppressed non‐radiative recombination. Based on the TRPL results, we constructed differential lifetime curves (Figure S6c). In the initial stage, the target film exhibits a steeper rise compared to the control film, reflecting suppressed rapid carrier trapping at interfaces and confirming effective passivation of surface defects. In the later stage, the differential lifetime of the target film continues to increase and remains consistently higher than that of the control, primarily attributable to reduced bulk defect density and overall improved film quality [37].

To investigate the out‐of‐plane optoelectronic uniformity of the films, PL measurements were performed from both the top and buried surfaces (Figure 2g,h). The control perovskite film exhibits distinct emission peaks at 739 and 732 nm on the top and buried surfaces, corresponding to organic‐rich and Cs‐rich regions, respectively, indicating the presence of vertical phase segregation. In contrast, the target perovskite film displays identical emission peaks at 738 nm on both surfaces, demonstrating that [SO_3_H‐PMIm][OTf] homogenizes the A‐site cation distribution along the thickness direction and suppresses Cs aggregation [38].

Grazing‐incidence X‐ray diffraction (GIXRD) was further employed to investigate the structural variations of the perovskite films at the top surface. In the control perovskite film, as the grazing incidence angle increases from 2° to 6°, the (100) diffraction peak shifts toward lower angles (Figure 2i,j and Figure S7a), indicating out‐of‐plane structural variations. In contrast, the target film exhibits a negligible shift in the peak position. The residual stress within the films was further evaluated by analyzing the slope of the 2*θ*‐sin^2^ω fitting curves. As shown in Figure S7b, the linear fitting of 2*θ*‐sin^2^ω yields slopes of −2.34 and −0.89 for the control and target films, respectively, indicating that the tensile stress in the target perovskite film is effectively relieved. The vertical shift of the (100) diffraction peak in the control film is attributed to internal strain arising from lattice mismatch, reflecting out‐of‐plane structural inhomogeneity. Owing to the radius disparity between MA/DMA^+^ and Cs^+^, phase separation between MA/DMA‐rich and Cs‐rich domains occurs along the thickness direction in the control film, forming a compositional gradient ranging from an organic‐rich surface to a Cs‐rich bottom, thereby inducing out‐of‐plane lattice mismatch. The incorporation of [SO_3_H‐PMIm][OTf] effectively suppresses this phenomenon [18]. To directly probe the vertical Cs distribution, XPS measurements on both the top surface and the buried interface of the perovskite films were performed. As shown in Figure 2k,l, the Cs 3d spectra of the target film exhibit negligible difference between the top surface and the buried interface, indicating a homogeneous vertical Cs distribution. In contrast, the control film shows a significantly stronger Cs 3d signal at the buried interface than at the top surface, revealing Cs accumulation at the bottom. This direct compositional evidence confirms that the incorporation of [SO_3_H‐PMIm][OTf] effectively suppresses Cs segregation along the film thickness direction, which is consistent with the conclusions drawn from top/buried PL and GIXRD analyses. The homogenized vertical distribution of A‐site cations is expected to reduce lattice mismatch and internal strain, thereby improving carrier transport and device stability.

Collectively, the control perovskite film exhibits severe compositional inhomogeneity in both in‐plane and out‐of‐plane directions. In‐plane, it shows random distribution and local enrichment of Cs and MA/DMA, while out‐of‐plane, it forms vertical phase segregation ranging from an organic‐rich surface to a Cs‐rich bottom, leading to lattice mismatch and internal strain. In contrast, upon introduction of [SO_3_H‐PMIm][OTf], the in‐plane compositional distribution of the film becomes homogeneous, with significantly enhanced and uniformly distributed carrier lifetimes and photoluminescence intensities. Simultaneously, the out‐of‐plane gradient distribution of A‐site cations is effectively suppressed, resulting in consistent optoelectronic properties at both the top and bottom surfaces. These results collectively demonstrate that [SO_3_H‐PMIm][OTf] achieves comprehensive homogenization of the perovskite film across both in‐plane and out‐of‐plane dimensions through regulating cation distribution (Figure 2m).

**FIGURE 3:**
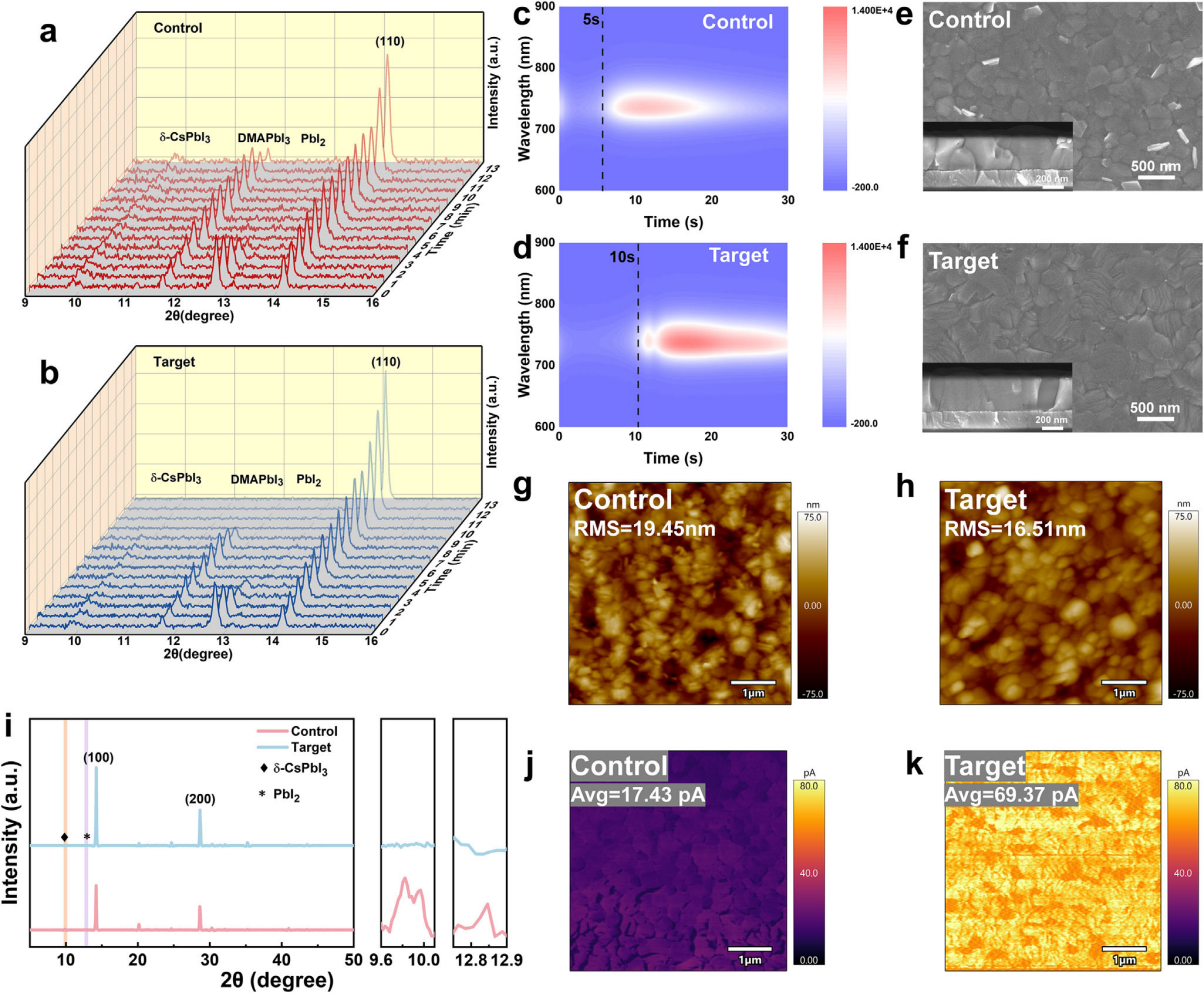
In situ XRD patterns of the (a) control and (b) target perovskite films during the annealing process. In situ PL spectra of the (c) control and (d) target perovskite films during the annealing process. (e,f) Top‐view SEM images (inset: cross‐sectional SEM images), (g,h) AFM images, (i) XRD patterns, (j,k) c‐AFM images of the control and target perovskite films.

In situ X‐ray diffraction (XRD) was performed to monitor the crystallization sequence during annealing at 100°C (Figure 3a,b). During the first 4 min, both control and target films show similar trends: PbI_2_ decreases, while the α‐phase and DMAPbI_3_ peaks intensify. After 4 min, a clear divergence occurs. In the target film, the DMAPbI_3_ peak declines rapidly and disappears completely by 10 min, with the δ‐CsPbI_3_ peak concurrently vanishing. In stark contrast, the control film exhibited a sluggish and incomplete transformation. Even after extending the annealing time to 14 min, both the DMAPbI_3_ intermediate phase and the δ‐CsPbI_3_ phase remained clearly detectable. This stark difference underscores that the incorporation of [SO_3_H‐PMIm][OTf] effectively accelerates the complete conversion from the intermediate phases to the desired black‐phase perovskite, while simultaneously suppressing the formation of the photoinactive δ‐CsPbI_3_. More importantly, in the target film, the DMAPbI_3_ intermediate and δ‐CsPbI_3_ disappear concurrently (within ∼10 min), providing direct evidence that the organic cations and Cs^+^ enter the perovskite lattice without a detectable time lag, confirming that [SO_3_H‐PMIm][OTf] enables synchronous crystallization of all A‐site cations.

To elucidate the role of [SO_3_H‐PMIm][OTf] in crystallization, in situ PL spectroscopy was employed, resolving three distinct stages during film formation: initial PL rise (nucleation), PL quenching (solvent evaporation and grain boundary formation), and subsequent PL increase (Ostwald ripening and grain boundary reduction). As shown in Figure 3c,d, the target film exhibits a slower crystallization rate and stronger PL emission intensity. This is attributed to the reduction in large colloids due to suppressed precursor aggregation, which leads to fewer nucleation sites and lower nucleation density in the wet film, thereby slowing crystal growth and ensuring consistent crystallization kinetics. These results demonstrate the ability of [SO_3_H‐PMIm][OTf] to modulate crystallization, yielding perovskite films with large, well‐aligned grains [26, 38, 39].

The morphological evolution of the perovskite films incorporating [SO_3_H‐PMIm][OTf] was investigated using scanning electron microscopy (SEM) and atomic force microscopy (AFM). The top‐view SEM images, shown in Figure 3e,f, reveal that the control film exhibits numerous PbI_2_ precipitates. In contrast, the target film displays enlarged grain sizes and is free of PbI_2_ precipitates. Furthermore, cross‐sectional SEM images (insets of Figure 3e,f) indicate that, compared to the control film with multiple grain stackings, the target film forms a distinct monolithic grain arrangement, which favorably accelerates charge transfer. AFM analysis also confirms the increased grain size and absence of PbI_2_ precipitates in the target film. Additionally, the root‐mean‐square (RMS) roughness of the perovskite film decreases from 19.45 to 16.51 nm (Figure 3g,h). The larger grains and smoother perovskite surface facilitate better interfacial contact and more efficient charge transport.

The crystallinity and phase purity of the perovskite films were analyzed using XRD (Figure 3i). The results reveal that the diffraction peak intensities of the target film are significantly higher than those of the control film, indicating enhanced crystallinity, which is beneficial for charge transport. Notably, in the perovskite film without [SO_3_H‐PMIm][OTf], in addition to the (100) and (200) diffraction peaks attributed to the black cubic phase, diffraction peaks corresponding to the yellow δ‐phase (9.8°) are also observed. This suggests incomplete crystallization and sluggish kinetics of Cs‐based perovskites, leading to inhomogeneous distribution of Cs/MA/DMA components within the pure‐iodide WBG perovskite film. Upon introduction of [SO_3_H‐PMIm][OTf], the crystallization temperature of Cs‐based perovskites is significantly reduced, and the δ‐phase diffraction peaks are virtually absent, indicating more complete phase transformation and improved crystallization quality [22]. Furthermore, the presence of PbI_2_ at around 12.8° in the control film is also observed, which is consistent with the observations from SEM and AFM. To quantify lattice distortion, the Williamson–Hall (W–H) method was employed to analyze microstrain. The control film exhibits a microstrain of 1.35 × 10^−3^, whereas that in the target film significantly decreases to 1.24 × 10^−3^ (Figure S8). The release of lattice microstrain in the target film can reduce charge recombination centers and enhance carrier mobility and lifetime [40].

To investigate the conductive properties of the films, conductive atomic force microscopy (c‐AFM) measurements were performed under identical bias voltage, along with longitudinal conductivity measurements (Figure 3j,k and Figure S9). The average current of the target film increases from 17.43 to 69.37 pA, accompanied by a significant enhancement in conductivity. This improvement is attributed to the enhanced crystallinity of the (100) perovskite phase and reduced defect density, which facilitate charge transport and consequently yield a higher fill factor (FF) [41]. As shown in Figure S10a, the target film exhibits significantly enhanced absorption intensity compared to the control film. Tauc plot analysis confirms that both films maintain the same bandgap of 1.68 eV, indicating that [SO_3_H‐PMIm][OTf] does not alter the bandgap. Furthermore, the Urbach energy calculated from the absorption spectra decreases from 28.56 to 27.02 meV (Figure S10b), indicating reduced band‐edge energetic disorder and lower trap density in the target film as a result of its improved film quality [42].

**FIGURE 4:**
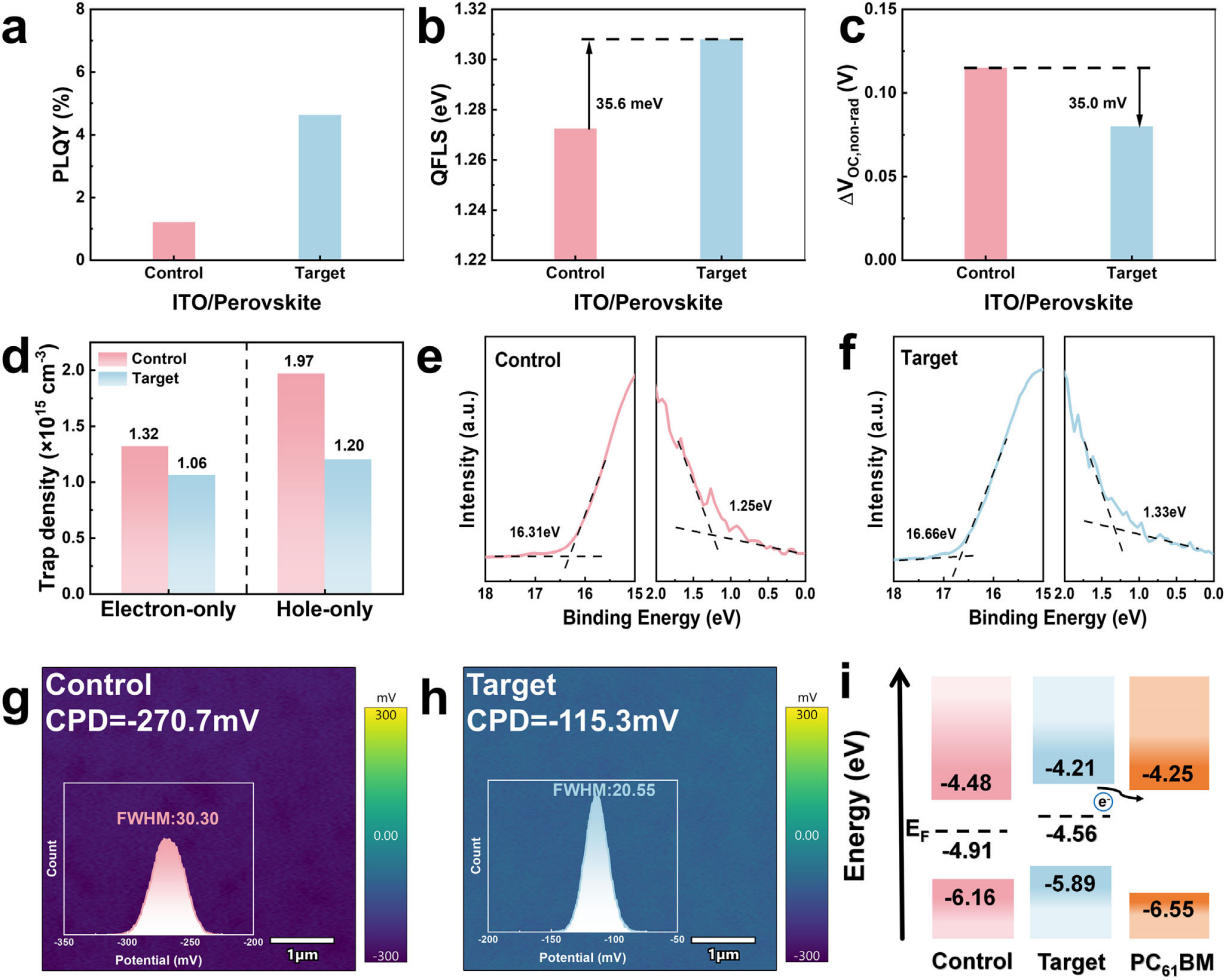
(a) PLQY and (b) QFLS; (c) nonradiative recombination *V*_OC_ calculated by the PLQY of the control and target perovskite films. (d) Trap densities of the hole‐only and electron‐only devices measured via the SCLC model. (e,f) UPS spectra and (g,h) KPFM images (inset is the potential distribution) of control and target perovskite films. (i) Energy level diagram of perovskite layer and PC_61_BM layer.

To quantify non‐radiative recombination losses, the photoluminescence quantum yield (PLQY) of the perovskite films was measured to estimate the quasi‐Fermi level splitting (QFLS). The corresponding PLQY and QFLS values are presented in Figure 4a,b, respectively. Compared to the control film, which exhibits a QFLS value of 1.272 eV, the target film shows a significantly improved QFLS of 1.308 eV. The non‐radiative voltage loss (Δ*V*_OC, non‐rad_) of the target film is calculated to be 79.9 mV, lower than that of the control film (114.9 mV) (Figure 4c), indicating reduced non‐radiative recombination in the target perovskite film [43, 44]. Space‐charge‐limited current (SCLC) measurements reveal that the corresponding trap density (*N*_t_) decreases from 1.32 × 10^15^ to 1.06 × 10^15^ cm^−3^ for electron‐only devices, and from 1.97 × 10^15^ to 1.20 × 10^15^ cm^−3^ for hole‐only devices (Figure 4d and Figure S11). These results confirm that both hole and electron trap densities are substantially reduced in the target perovskite film, thereby mitigating non‐radiative recombination losses, which directly correlates with the improved crystal quality observed in preceding structural analyses [45].

Considering the potential influence of [SO_3_H‐PMIm][OTf] on the electronic structure of the perovskite films, ultraviolet photoelectron spectroscopy (UPS) measurements were performed, as shown in Figure 4e,f. The work function (WF), calculated from the secondary electron cutoff, decreases from 4.85 to 4.54 eV upon [SO_3_H‐PMIm][OTf] modification. Kelvin probe force microscopy (KPFM) analysis corroborates the UPS findings, revealing that the target perovskite film exhibits a larger average contact potential difference (CPD) compared to the control film, thereby validating the reduction in WF (Figure 4g,h). Furthermore, the CPD distribution of the target film is more concentrated than that of the control film (insets of Figure 4g,h), with the full width at half maximum (FWHM) decreasing from 30.30 to 20.55. This indicates that the addition of [SO_3_H‐PMIm][OTf] yields a uniform perovskite surface with superior electronic properties. The resulting energy level diagrams of the perovskite and electron transport layer (ETL) are depicted in Figure 4i. For the control and target films, the valence band maximum (VBM) is located 1.25 and 1.33 eV below the Fermi level (*E*_F_), respectively, indicating enhanced n‐type character in the target perovskite film. A plausible explanation is that the [SO_3_H‐PMIm][OTf] modification eliminates excess PbI_2_ (which acts as a p‐type semiconductor) at the perovskite surface while simultaneously forming an interfacial dipole. Moreover, the conduction band minimum (CBM) of the target film aligns more closely with that of the ETL at the n‐type contact interface, thereby enabling more efficient carrier extraction and transport at the perovskite/ETL interface [46].

**FIGURE 5:**
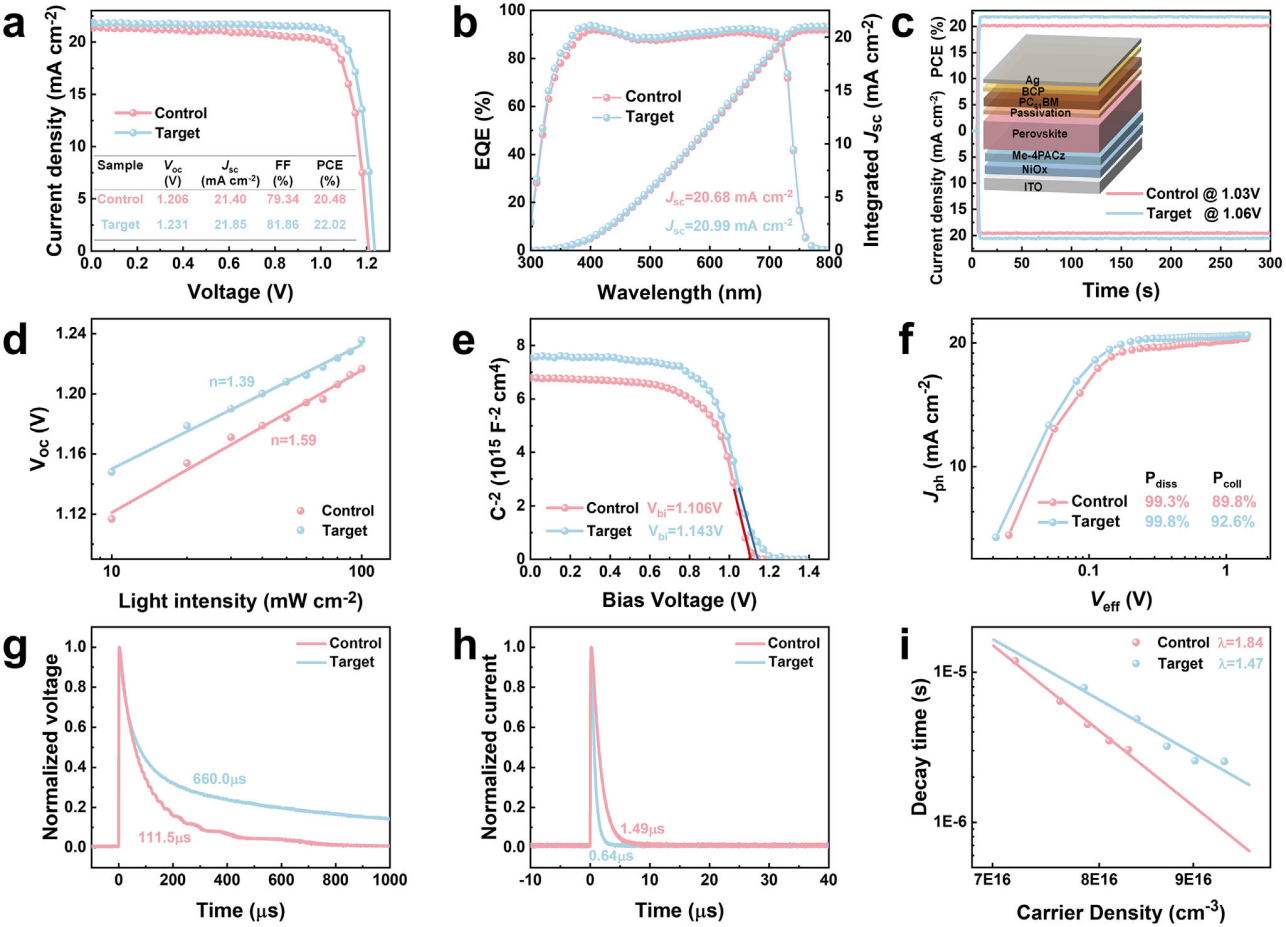
(a) *J–V* curves and (b) EQE spectra and integrated current densities of the control and target WBG‐PSCs. (c) Stabilized power output and current density at the MPP for the control and target WBG‐PSCs. (d) *V*_OC_ values of control and target WBG‐PSCs as a function of light intensity. (e) Mott–Schottky plots, (f) *J*_ph_‐*V*_eff_ curves of the control and target WBG‐PSCs. (g) TPV, and (h) TPC of control and target WBG‐PSCs. (i) The TPV decay time is a function of carrier density intensity.

To investigate the effect of [SO_3_H‐PMIm][OTf] concentration on device performance, WBG‐PSCs were fabricated, with optimal performance achieved at 0.2 mg mL^−1^ and excellent reproducibility (Figure S12). The control device achieved a power conversion efficiency (PCE) of 20.48%, with a *V*_OC_ of 1.206 V, short‐circuit current density (*J*_SC_) of 21.40 mA cm^−2^, and FF of 79.34%, while the target device delivered a champion PCE of 22.02%, with a *V*_OC_ of 1.231 V, *J*_SC_ of 21.85 mA cm^−2^, and FF of 81.86% (Figure 5a and Table S2). The hysteresis index was substantially reduced from 6.6% to 2.5%. Integrated *J*_SC_ values from external quantum efficiency (EQE) spectra (20.68 and 20.99 mA cm^−2^ for control and target devices, respectively) agreed well with current density‐voltage (*J‐V*) measurements (Figure 5b). Maximum power point tracking under continuous illumination demonstrated stable power output over 300 s for the target device (Figure 5c). The pure‐iodide WBG‐PSCs reported in the literature have been summarized in Table S3.

To elucidate the underlying mechanism, electrochemical impedance spectroscopy (EIS) was performed under dark conditions at 0.8 V (Figure S13). The components *R*_s_, *R*_ct_, and *R*_rec_ represent the internal series resistance, charge transfer resistance, and recombination resistance, respectively, and the fitted parameters are detailed in Table S4. The target WBG‐PSC exhibits a decrease in both *R*_s_ and *R*_ct_, while *R*_rec_ shows a significant increase. The decreased *R*_ct_ indicates more efficient charge extraction at interfaces, while the increased *R*_rec_ directly evidences suppressed non‐radiative recombination, collectively leading to the enhanced *V*_OC_. Light intensity–dependent *J–V* measurements (Figure 5d and Figure S14) revealed ideality factors (n) of 1.59 and 1.39 for the control and target devices, respectively, with the smaller n value suggesting suppressed trap‐assisted recombination. The fitting slope α for *J*_SC_ as a function of light intensity increased from 0.992 to 0.995, approaching unity and indicating more efficient charge collection. Mott–Schottky analysis (Figure 5e) further showed an increased built‐in potential (*V*_bi_) from 1.106 to 1.143 V, which enhances charge extraction and suppresses recombination, collectively accounting for the improved *V*_OC_ [47].

The enhancement of the built‐in electric field not only suppresses recombination but is also reflected in the dark behavior of the devices. The dark *J–V* curves reveal that the target device exhibits a substantially reduced dark saturation current compared to the control device (Figure S15), indicating suppressed non‐radiative recombination pathways and improved charge collection efficiency, thereby contributing to enhanced *V*_OC_ and *J*_SC_. To further investigate the charge collection efficiency of the WBG‐PSCs, photocurrent density (*J*_ph_) as a function of effective internal voltage (*V*_eff_) measurements were conducted (Figure 5f). For the target device, the calculated exciton dissociation efficiency (*P*_diss_) and charge collection efficiency (*P*_coll_) are 99.8% and 92.6%, respectively, compared to 99.3% and 89.8% for the control device. The enhanced *P*_diss_ and *P*_coll_ values in the target device demonstrate that the incorporation of [SO_3_H‐PMIm][OTf] effectively promotes charge generation and collection, thereby improving the photovoltaic performance [48].

Based on the analysis above, the target device exhibits superior suppression of recombination through reduced defect density, optimized energy level alignment, and enhanced carrier dynamics. The losses in *V*_OC_ and FF were systematically analyzed (Figure S16). Given the identical bandgap of both devices, their radiative recombination losses are comparable. The target WBG‐PSCs shows reduced non‐radiative recombination loss in the perovskite absorption layer, while improved energy level alignment minimizes interfacial non‐radiative recombination. For FF loss, the target device shows a slightly reduced contribution from non‐radiative recombination, while the loss from charge transport limitations is markedly diminished due to improved charge extraction and transport [49].

Analysis of the series resistance (*R*_s_) and reverse saturation current (*J*_0_) further corroborates the aforementioned loss mechanisms and provides critical insights into the performance enhancement of the WBG‐PSCs (Figure S17). The series resistance decreases from 0.86 Ω cm^2^ for the control device to 0.72 Ω cm^2^ for the target device. A lower *R*_s_ minimizes resistive losses and facilitates more efficient charge extraction, thereby contributing to the improved FF and PCE. The reverse saturation current values for the control and target devices are 6.70 × 10^−9^ and 4.63 × 10^−10^ mA cm^−2^, respectively. The diminished *J*_0_ indicates reduced non‐radiative recombination, which directly accounts for the enhanced open‐circuit voltage and further contributes to the improved photovoltaic performance [50].

Transient photovoltage (TPV) and transient photocurrent (TPC) measurements were performed to verify the changes in carrier recombination and transport. As shown in Figure 5g,h, the target device exhibits a significantly prolonged charge recombination lifetime, increasing from 111.5 to 660.0 µs, and a shortened charge transport time, decreasing from 1.49 to 0.64 µs, indicating effective suppression of recombination and reduced defect density. Light intensity‐dependent TPV measurements (Figure S18) reveal λ values of 1.84 and 1.47 for the control and target devices, respectively (Figure 5i), where a lower λ signifies a transition from trap‐assisted to bimolecular recombination. These results confirm reduced defect density and suppressed non‐radiative recombination, contributing to the enhanced photovoltaic performance [51, 52].

**FIGURE 6:**
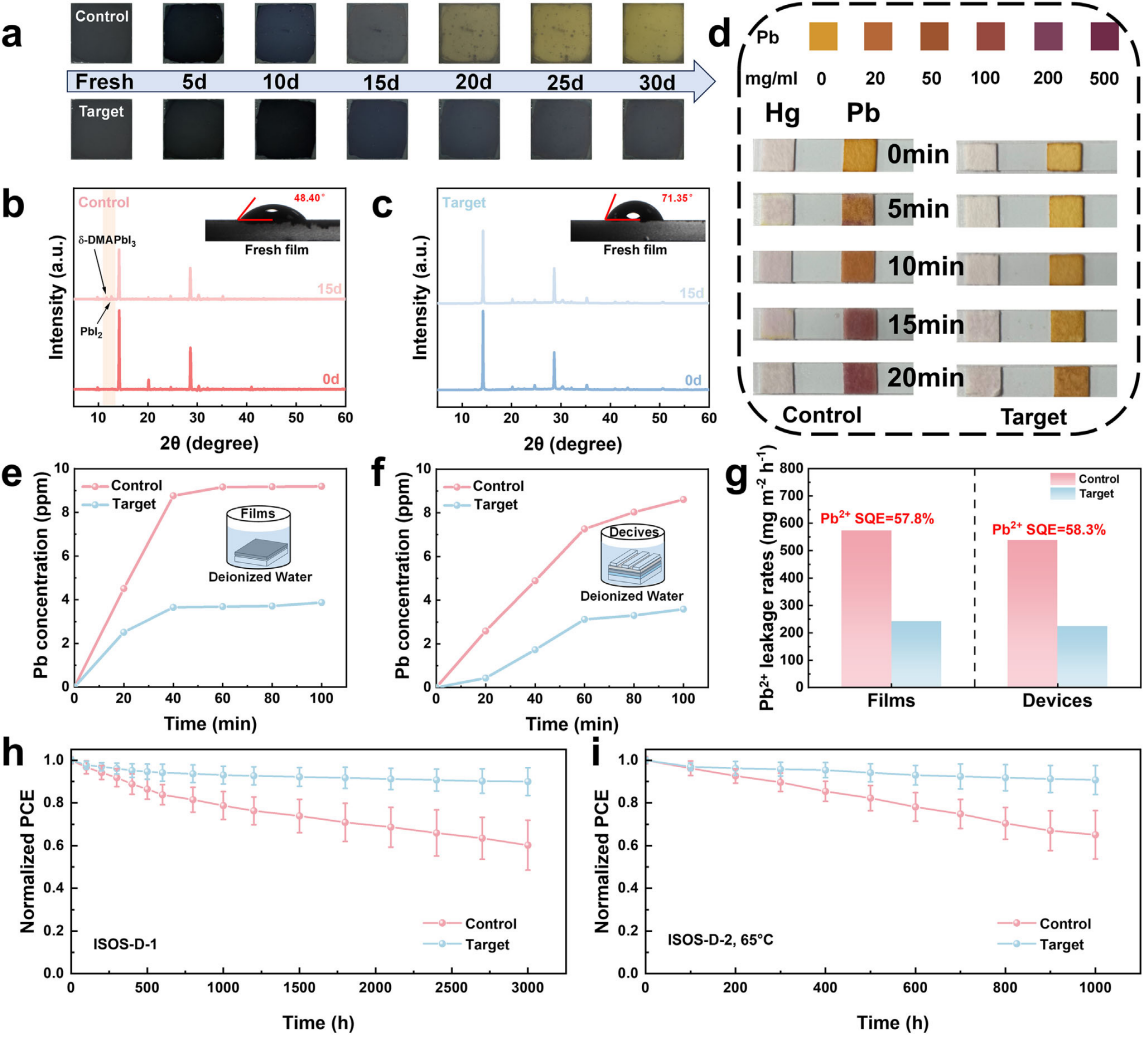
(a) The evolution of photographs of perovskite films under conditions of room temperature and 60% RH storage for 30 days. (b,c) XRD patterns of control and target films before and after 15 days of aging at room temperature and 60% RH. (d) Images of the Pb‐indicator papers. (e,f) Time‐dependent Pb concentration in 50 mL of deionized water (25°C, pH 7) for control and target (e) perovskite films and (f) WBG‐PSCs (two pieces per sample, 2 cm × 2 cm each). (g) Comparison of Pb^2+^ leakage rates for both control and target films, as well as their corresponding WBG‐PSCs, after 100 min exposure to water (pH 7). (h) The long‐term stability of unencapsulated WBG‐PSCs in ambient air with 20% ± 5% RH at room temperature according to ISOS‐D‐1. (i) The thermal stability of unencapsulated WBG‐PSCs in ambient air with 20% ± 5% RH at 65°C according to ISOS‐D‐2. Error bars represent standard deviation (SD) from five independent samples (n = 5).

The intrinsic ecological risks associated with lead toxicity in perovskite materials remain a critical obstacle to their commercial application. Finally, we enhanced the practical applicability of WBG‐PSCs in terms of long‐term stability and lead leakage. As shown in Figure 6a, when stored under conditions of 60% relative humidity (RH) at room temperature (RT), the control film gradually faded and completely turned from black to yellow after 20 days, whereas the perovskite film incorporating [SO_3_H‐PMIm][OTf] exhibited no obvious color change, indicating improved tolerance to harsh environments. The mechanism underlying lattice stabilization is undoubtedly attributable to superior film quality and fluorine‐induced hydrophobicity, as evidenced by the increased water contact angle from 48.40° to 71.35° (insets of Figure 6b,c). Tracking the evolution of XRD patterns (Figure 6b,c), the control film exhibited a significantly weakened (001) peak of the cubic perovskite at 14.2°, along with emerging peaks of PbI_2_ and δ‐DMAPbI_3_, indicating severe lattice degradation upon continuous aging. In contrast, upon addition of [SO_3_H‐PMIm][OTf], the (001) peak intensity remained essentially unchanged, and no PbI_2_ or δ‐DMAPbI_3_ phases were observed.

In addition to the stabilization of the lattice, the strong interaction between ─SO_3_^−^ and Pb^2+^ also prevents lead leakage, mitigating environmental impact. As shown in Figure S19, upon direct immersion of eight perovskite films (each 2 cm × 2 cm, thickness ∼530 nm, corresponding to a total Pb content of ∼280 µg per film) into 100 mL of deionized water, all films immediately turned yellow due to the vigorous decomposition of the perovskite structure. However, after 20 min, the control films had clearly dissolved and became transparent, while the yellow PbI_2_ remained in the target films, indicating suppressed lead leakage. To assess lead leakage levels, the variation in lead concentration in the aqueous solution with immersion time was monitored using lead indicator paper. Compared with the control sample, where the color of the indicator paper turned purple within 20 min, the target sample exhibited only a slight color change, implying a substantially lower lead concentration (Figure 6d) [53]. To better quantify lead leakage levels, inductively coupled plasma mass spectrometry (ICP‐MS) was employed to measure the lead concentration after immersing two control and target perovskite film, as well as the corresponding WBG‐PSCs (each 2 cm × 2 cm, thickness ∼530 nm, corresponding to a total Pb content of ∼280 µg per film) individually in 50 mL of deionized water at 25°C and pH 7. As shown in Figure 6e,f, within 100 min, the lead concentrations in the control film and device reached 9.19 and 8.61 ppm, respectively, due to water infiltration and rapid dissolution of lead compounds. In contrast, the target film and device exhibited lead concentrations reduced to 3.87 and 3.59 ppm, corresponding to lead sequestration efficiencies of 57.8% and 58.3%, respectively (Figure 6g) [54]. To evaluate the reproducibility, parallel leaching experiments were conducted on five independent pairs of films and devices under identical conditions over a duration of 120 min. The average Pb^2+^ concentration in the leachate from the soaked films decreased from 12.77 ppm (control) to 5.43 ppm (target), while that from the soaked devices decreased from 10.33 ppm (control) to 5.22 ppm (target). These results consistently confirm the effective suppression of lead leakage achieved by the [SO_3_H‐PMIm][OTf] modification (Figure S20). This significant improvement can be attributed to the strong interaction between ─SO_3_^−^ in [SO_3_H‐PMIm][OTf] and Pb^2+^ in the perovskite films, which stabilizes the perovskite lattice structure. Additionally, the inherent hydrophobicity of the ─CF_3_ functional group provides synergistic protection against water ingress. Collectively, these effects substantially suppress moisture‐induced perovskite degradation and Pb^2+^ migration, ultimately leading to effective mitigation of lead leakage.

In terms of device durability, the efficiency evolution of the control and target WBG‐PSCs under various conditions was monitored. Dark storage stability tests under ambient conditions (RT, 20% ± 5% RH, ISOS‐D‐1 protocol) showed that the target WBG‐PSCs retained 90.00% of its initial PCE after 3000 h, significantly higher than the control WBG‐PSCs, which retained only 60.20% (Figure 6h). Thermal stability tests (65 °C, 20% ± 5% RH, ISOS‐D‐2 protocol) further confirmed this advantage. The target WBG‐PSCs retained 90.78% of their initial PCE after continuous heating at 65°C for 1000 h, whereas the control WBG‐PSCs retained only 65.10% (Figure 6i), demonstrating excellent tolerance to external thermal stress. This enhanced stability is attributed to the homogenized distribution of A‐site cations achieved by [SO_3_H‐PMIm][OTf], which suppresses cation phase segregation and lattice distortion, both of which are key factors that typically compromise device durability. Operational photostability tests under continuous 1‐sun illumination in ambient air at room temperature (ISOS‐L‐1 protocol) further demonstrated the superior durability of the target devices, which retained ∼91% of their initial PCE after 1000 h, while the control devices retained only ∼70% under identical conditions (Figure S21). Moreover, consistent with previous reports, the pure‐iodide WBG‐PSCs inherently exhibit excellent photostability, effectively suppressing photoinduced halide segregation [47]. The pure‐iodide WBG‐PSCs incorporating [SO_3_H‐PMIm][OTf] combine high efficiency, excellent stability, and enhanced safety, thus holding great promise for application in high‐efficiency and stable tandem devices.

## 3 Conclusions

In summary, we have developed a multifunctional IL, [SO_3_H‐PMIm][OTf], to address the critical challenges of A‐site cation inhomogeneity and lead toxicity in pure‐iodide WBG‐PSCs. The additive simultaneously regulates precursor chemistry and crystallization kinetics: it coordinates with Cs^+^ via the sulfonate group to increase CsI solubility and lower the crystallization temperature of Cs‐based perovskites, anchors MA/DMA cations via hydrogen bonding, and coordinates with undercoordinated Pb^2+^ ions through the sulfonate group. These synergistic interactions suppress colloidal aggregation and enable synchronous crystallization of Cs‐ and organic‐based components, achieving homogeneous A‐site cation distribution across both in‐plane and out‐of‐plane dimensions. The resulting Cs_0.3_DMA_0.2_MA_0.5_PbI_3_ pure‐iodide WBG‐PSCs deliver a champion efficiency of 22.02% with an *V*_OC_ of 1.231 V and retain 90% of initial efficiency after 3000 h under the ISOS‐D‐1 protocol, while reducing lead leakage by over 58%. This integrated molecular design not only provides a versatile strategy for overcoming cation inhomogeneity and lead toxicity but also establishes a new paradigm for developing efficient, stable, and environmentally benign perovskite photovoltaics, with significant implications for tandem device applications.

## Author Contributions

**Kai Wu**: writing – original draft, conceptualization, investigation, formal analysis, data curation, methodology. **Lei Yang**: investigation, data curation, formal analysis. **Guoqing Du**: formal analysis, investigation. **Xin Wang**: investigation. **Jiali Wei**: formal analysis. **Tiantian Li**: supervision. **Pengfei Zhang**: supervision, resources. **Xiaodan Zhang**: supervision, resources. **Fuhua Hou**: supervision, resources, writing – review and editing, funding acquisition.

## Conflicts of Interest

The authors declare no conflict of interest.

## Supporting information




**Supporting file**: advs77993‐sup‐0001‐SuppMat.docx.

## Data Availability

The data that support the findings of this study are available from the corresponding author upon reasonable request.
