# Supplementary material for: Multifunctional Ionic Liquid Enabling Cation Homogenization and Lead Chelation for Pure‐Iodide Wide‐Bandgap Perovskite Solar Cells
Source: Adv Sci (Weinh). 2026 Sep 27:e77993. Online ahead of print. doi: 10.1002/advs.77993 (PMC13616288; doi:10.1002/advs.77993)
Supplement: Supplementary file 1 [file ADVS-9999-e77993-s001.docx]

Supporting Information

**Multifunctional Ionic Liquid Enabling Cation Homogenization and Lead Chelation for Pure-Iodide Wide-Bandgap Perovskite Solar Cells**

Kai Wu, Lei Yang, Guoqing Du, Xin Wang, Jiali Wei, Tiantian Li, Pengfei Zhang*, Xiaodan Zhang*, and Fuhua Hou*

K. Wu, L. Yang, G. Du, X. Wang, T. Li, F. Hou

Key Laboratory of Semiconductor Photovoltaic Technology and Energy Materials of Inner Mongolia Autonomous Region, School of Physical Science and Technology, Inner Mongolia University, Hohhot 010021, China

E-mail: houfuhua@imu.edu.cn

J. Wei, X. Zhang

Institute of Photoelectronic Thin Film Devices and Technology, Tianjin Key Laboratory of Efficient Utilization of Solar Energy, State Key Laboratory of Photovoltaic Materials and Cells, Nankai University, Tianjin 300350, China

E-mail: xdzhang@nankai.edu.cn

P. Zhang

Institutes of Biomedical Sciences, School of Life Sciences, Inner Mongolia University, Hohhot 010070, China

E-mail: zhangpengfei@imu.edu.cn

F. Hou

Research Center for Quantum Physics and Technologies, Inner Mongolia University, Hohhot 010021, China

Experimental Section

**Materials**

Nickel nitrate hexahydrate (Ni(NO_3_)_2_·6H_2_O, ≥98%) was purchased from Sinopharm Chemical Reagent Co., Ltd. Deionized water (DI), lead (II) acetate trihydrate (PbC_4_H_6_O_4_·3H_2_O, 99.5-102.0%) and dimethyl sulfoxide (DMSO, ≥99.9%) were purchased from Sigma-Aldrich. Tetramethylammonium hydroxide pentahydrate (TMAOH, 97%), isopropyl alcohol (IPA, ≥99.9%), N, N-dimethylformamide (DMF, 99.8%) were obtained from Aladdin Reagent. Ammonium methyl chloride (MACl, 99.9%), dimethylammonium iodide (DMAI, 99.9%), methylammonium iodide (MAI, 99.9%), cesium iodide (CsI), lead iodide (PbI_2_, 99.999%), and [6,6]-phenyl-C_61_-butyrate methyl ester (PC_61_BM) were purchased from Advanced Election Technology. Lead (II) chloride(PbCl_2_, 99.9%), [4-(3,6-dimethyl-9H-carbazol-9yl) butyl] phosphonic acid (Me-4PACz, 99%), 2-phenylethylamine hydroiodide (PEAI, ≥99.5%) and 2,9-dimethyl-4,7-diphenyl-1,10-phenanthroline (BCP) were bought from Xi’an Yuri Solar Co., Ltd. Ag-G5036 (99.999%) was purchased from Beijing Dream Material Technology Co., Ltd. 1-Propylsulfonate-3-methylimidazolium trifluoromethanesulfonate ([SO_3_H-PMIm][OTf], ≥99%) was purchased from Lanzhou Greenchem ILs. Indium tin oxide (ITO) conductive glass substrates measuring 2 cm × 2 cm were purchased from You Xuan Ltd.

**Synthesis of NiO_x_ nanoparticles and preparation of NiO_X_ suspensions**

The NiO_x_ precursor solution was synthesized via a sol-gel method. Briefly, a stoichiometric amount of Ni(NO_3_)_2_·6H_2_O was dissolved in deionized water, and the resulting solution was gradually added dropwise into an aqueous solution of TMAOH under continuous stirring. The pH of the mixture was adjusted to 10, yielding an apple-green precipitate. The product was then dried and calcined in air at 270 °C for 2 h to decompose into dark black NiO_x_ nanocrystals. Finally, the obtained NiO_x_ nanoparticles were dispersed in a mixed solvent of IPA and DI (IPA: DI = 1:2, v: v) at a concentration of 15 mg mL^-1^ for further use.

**Device fabrication**

ITO-coated glass substrates were sequentially cleaned via sonication in detergent, deionized water, and ethanol. The substrates were subsequently treated with UV-ozone for 20 min to enhance surface wettability. The NiO_x_ HTL was deposited by spin-coating the NiO_x_ nanocrystal ink (15 mg mL^-1^) at 4000 rpm for 30 s, followed by thermal annealing in air at 150 °C for 20 min. The resulting films were then transferred into a nitrogen-filled glovebox for further processing. Next, a solution of Me-4PACz (0.5 mg mL^-1^ in IPA) was spin-coated onto the NiO_x_ film at 5000 rpm for 30 s and annealed at 100 °C for 10 min to form the subsequent functional layer.

For perovskite precursor preparation, 1.2 M control Cs_0.3_DMA_0.2_MA_0.5_PbI_3_ perovskite precursor solution was prepared by mixing two precursors with a volume ratio of 1:1. The precursor Ⅰ was prepared by mixing MAI, Pb (Ac)_2_·3H_2_O and PbCl_2_ with a molar ratio of 3:0.85:0.15 in DMF. The precursor Ⅱ was prepared by mixing CsI, DMAI and PbI_2_ with a molar ratio of 0.6:0.4:1 in DMF/DMSO (19:1, v: v). The two solutions were stirred at room temperature for 3 h before mixing. After mixing, the target solution was stirred for 30 min before use. For Cs_0.3_DMA_0.2_MA_0.5_PbI_3_ with [SO_3_H-PMIm][OTf], the [SO_3_H-PMIm][OTf] was added into the Cs_0.3_DMA_0.2_MA_0.5_PbI_3_ perovskite precursor and stirred for 30 min before use.

For Cs_0.3_DMA_0.2_MA_0.5_PbI_3_ perovskite films fabrication, the perovskite precursor solution was spin-coated onto the prepared substrates at 4000 rpm for 40 s. The coated substrates were allowed to rest for 3 min at room temperature before thermal annealing. Subsequently, the films were annealed on a hotplate at 100 °C for 30 min.

Finally, the passivation layer was formed by spin-coating PEAI solution (2 mg mL^-1^ in IPA) atop the perovskite films at 5000 rpm for 30 s, followed by thermal annealing at 100 °C for 5 min. Next, the PC_61_BM was deposited on the perovskite films at 2500 rpm for 30 s and the BCP was deposited at 5000 rpm for 30s. Finally, the films were transferred to a vacuum evaporator for deposition of Ag (80 nm).

**Characterization**

Fourier transform infrared (FTIR) spectroscopy was performed on a VERTEX 70 spectrometer (Bruker). Proton nuclear magnetic resonance (^1^H NMR) spectra were recorded on a Bruker AVANCE III HD 400 spectrometer operating at 400 MHz. Raman mapping measurements were conducted on an ATR8800 system (Optosky (Xiamen) Photonics Co., Ltd.) with a 785 nm excitation laser to analyze the spatial distribution of vibrational modes in the samples. Steady-state photoluminescence (PL) and time-resolved photoluminescence (TRPL) spectra, along with their corresponding mapping, were measured using a PicoQuant MicroTime 100 confocal microscopic system (ETSC). Dynamic light scattering (DLS) measurements were performed on a Litesizer DLS 501 system (Anton Paar GmbH) to analyze the particle size distribution. Zeta potential measurements were performed using an NS-90Z Plus nanoparticle size and zeta potential analyzer (Zhuhai OMEC Instruments Co., Ltd.). Scanning electron microscopy (SEM) was performed using a Hitachi SU8010 microscope to examine the surface morphology of the samples. X-ray diffraction (XRD) measurements were conducted on a Bruker D8 diffractometer with Cu Kα radiation (λ = 1.5405 Å) over a 2θ range of 5° to 60° to analyze the crystal structure of the perovskite films. Steady-state photoluminescence (PL) spectra were recorded using an Edinburgh Instrument FLS980 fluorometer. Time-resolved photoluminescence (TRPL) measurements were carried out with a 475 nm excitation source to evaluate charge carrier dynamics. The electronic structure of the samples was investigated using X-ray photoelectron spectroscopy (XPS) and ultraviolet photoelectron spectroscopy (UPS), performed on a PHI ULVAC-5000 VP III system. In situ photoluminescence (PL) measurements were performed using an InView-PL online in situ fluorescence spectrometer. The absolute Photoluminescence quantum yield (PLQY) was measured using an LuQY Pro (LP20-32) system (Quantun yield Berlin GmbH). The samples were excited inside an integrating sphere by a 532 nm laser, spot size is 0.1cm^2^, whose intensity was equivalent to 1 sun condition. The measured PLQY was then used to calculate the quasi-Fermi level splitting (QFLS). Atomic force microscopy (AFM), Kelvin probe force microscopy (KPFM), and conductive atomic force microscopy (c-AFM) were performed on an Asylum Research Cypher S series system (Oxford Instruments) to characterize the surface morphology, surface potential, and local conductivity of the samples, respectively. UV-vis absorption spectra were recorded on a LAMBDA 750 spectrophotometer (PerkinElmer). The current density-voltage (*J-V*) characteristics of the wide-bandgap perovskite solar cells (WBG-PSCs) were measured under simulated AM 1.5G illumination (100 mW cm^-2^) using an SS-X50 solar simulator (Enli Technology Co., Ltd.). The light intensity was calibrated using a certified silicon reference cell (SRC-00266, Enli Technology). The measurements were performed with a Keithley 2400 source meter, employing a bidirectional voltage sweep from 1.8 V to -0.2 V (reverse scan) and back (forward scan) at a step size of 30 mV and a delay time of 10 ms. The overall size of the Wide-bandgap perovskite solar cells (WBG-PSCs) has refined as 4 cm^2^ (2 cm × 2 cm). Effective area of WBG-PSCs is refined to 0.24 cm^2^ and the aperture area when measured is 0.084 cm^2^. External quantum efficiency (EQE) spectra were acquired using a Newport QUANTX-300 system. Transient photocurrent (TPC) and transient photovoltage (TPV) measurements were conducted using a Tran PVC system (Oriental Spectra Technology). Mott-Schottky and electrochemical impedance spectroscopy (EIS) measurements were conducted using a CS360 electrochemical workstation (Wuhan Corrtest Instruments Corp., Ltd.). The contact angle measuring instrument (SDC-350, Dongguan Shengding Precision Instrument Co., Ltd. Dongguan, China) was used to drop 2 uL of deionized water to measure the water. Inductively coupled plasma mass spectrometry (ICP-MS) measurements were conducted on a Plasma MS 400 system (NCS Testing Technology Co., Ltd.) to determine the elemental composition of the samples.

**Supplementary Notes**

**Note S1: Time-resolved photoluminescence (****TRPL)**

Time-resolved photoluminescence (TRPL) was employed to probe the charge carrier recombination kinetics and molecular dynamics. The decay profiles were analyzed using a biexponential fitting function:^[1]^

$$\begin{aligned} y=A_{1}exp(-x/\tau_{1})+A_{2}exp(-x/\tau_{2})\#\left( S1 \right) \end{aligned}$$

Where *τ*_1_ is the extraction time of charge carriers at the interface, *τ*_2_ is the decay time generated by free charge carrier recombination, and *A*_1_, *A*_2_ represents the decay amplitude.

**Note S2: Williamson-Hall (W-H) analysis method**

The Williamson-Hall (W-H) analysis method to estimate the micro-strain in order to gain in-depth insights into the changes of the XRD peaks, specifically based on the following formula:^[2]^

$$\begin{aligned} \beta cos\theta=\varepsilon\left( 4sin\theta\right)+\frac{K\lambda}{D}\#\left( S2 \right) \end{aligned}$$

Where *β* is the full width at half maximum (FWHM) of the XRD peak, *θ* is the diffraction angle, *ε* is the micro-strain, *K* is the Scherrer constant, *λ* is the wavelength of the X-ray radiation, and *D* is the grain size.

**Note S3: Quasi-Fermi level splitting (QFLS) calculation**

QFLS is calculated by photoluminescence quantum yield (PLQY) using the following equation:

$$\begin{aligned} QFLS=k_{B}T\times ln(PLQY\times J_{G}/J_{0,rad})\#\left( S3 \right) \end{aligned}$$

Where *J*_G_ is the generated current density at one-sun illumination, it is approximated by the short-circuit current density of the complete solar cell. *J*_0,rad_ is the radiative recombination current in the dark, it can be calculated by the following equation:

$$\begin{aligned} J_{0,rad}=e\int_{0}^{\infty} {EQE}_{PV}(E) \phi_{BB}(E) dE\#\left( S4 \right) \end{aligned}$$

The estimation is derived by integrating the overlap between the external quantum efficiency (EQE) spectrum of the WBG-PSCs and the 300 K blackbody radiation spectrum over the energy range.

$$\begin{aligned} \phi_{BB}=\frac{2\pi E^{2}}{h^{3}c^{2}}\cdot\frac{1}{\exp\left( \frac{E}{k_{B}T} \right)-1}\#\left( S5 \right) \end{aligned}$$

Where *E* is the photon energy, *h* is the Plank constant, and *c* is the light speed in vacuum.^[3, 4]^

**Note S4: corresponding *V*_OC_ loss**

The corresponding *V*_OC_ loss caused by non-radiative recombination can be determined by the following equation:

$$\begin{aligned} \Delta V_{OC,non-rad}=-\frac{k_{B}T}{q}ln(PLQY)\#\left( S6 \right) \end{aligned}$$

In the equation, Δ*V*_OC,non−rad_ represents the *V*_OC_ loss due to non-radiative recombination, *k*_B_ is the Boltzmann constant, *T* is the temperature in Kelvin, and *q* is the elementary charge.^[5]^

**Note S5: Space charge limited current (****SCLC) analysis**

Space charge-limited current (SCLC) spectra ware conducted on the ITO/SnO_2_/perovskite/PC_61_BM/Ag device structure to evaluate the electron trap density (*n*_trap_) and charge carrier mobility (). The *n*_trap_ was calculated using the following equations:

$$\begin{aligned} n_{trap}={2\varepsilon\varepsilon_{0}V_{TFL}}/\left( ⅇL^{2} \right)\#\left( S7 \right) \end{aligned}$$

Where *e* is the elementary charge, *ε* represents the relative dielectric constant, *ε*_0_ represents the vacuum dielectric constant, and *L* is the thickness of the perovskite layer, and *V*_TFL_ is trap-filled limit voltage.^[6]^

**Note S6: Ultraviolet photoelectron spectroscopy (UPS) analysis**

According to the equation:

$$\begin{aligned} E_{F}=E_{cutoff}-h\nu\#\left( S8 \right) \end{aligned}$$

$$\begin{aligned} E_{VB}={E_{F}-E}_{VBM}\#\left( S9 \right) \end{aligned}$$

$$\begin{aligned} E_{CB}=E_{VB}+E_{g}\#\left( S10 \right) \end{aligned}$$

Where *hν*=21.22 eV, *E*_F_ is the fermi level, *E*_cutoff_ is the secondary electron cutoff edge. *E*_VBM_ is the intersection point between the tangent line of the steeply rising section in the UPS spectrum and the baseline. *E*_VB_ is the energy of the Valence Band Maximum, *E*_CB_ is the energy of the Conduction Band Minimum.

**Note S7:** **The variation of *V*_OC_ with light intensity**

The variation of *V*_OC_ with light intensity follows Equation:

$$\begin{aligned} V_{OC}=\frac{nk_{B}T}{q}ln(I)+C\#\left( S11 \right) \end{aligned}$$

Where *I* is the incident light intensity, *k*_B_ is the Boltzmann constant, *T* is the temperature (298 K), *n* is the ideality factor, and *q* is the elementary charge.

**Note S8: Photocurrent Density (*J*_ph_) – Effective Internal Voltage (*V*_eff_) Analysis**

The relationship between photocurrent density (*J*_ph_) and effective internal voltage (*V*_eff_) was employed to investigate the charge collection efficiency of the control and target devices. *J*_ph_ is calculated as:

$$\begin{aligned} J_{\mathrm{ph}}=J_{L}{- J}_{D}\#\left( S12 \right) \end{aligned}$$

where *J*_L_ and *J*_D_ represent the current densities under illumination and in the dark, respectively.

The charge collection probability (*P*_coll_) and exciton dissociation efficiency (*P*_diss_) are defined as:

$$\begin{aligned} P_{\mathrm{coll}}=J_{\mathrm{sc}}/J_{\mathrm{ph},\mathrm{sat}}\#\left( S13 \right) \end{aligned}$$

$$\begin{aligned} P_{\mathrm{diss}}=J_{\max}/J_{\mathrm{ph},\mathrm{sat}}\#\left( S14 \right) \end{aligned}$$

where *J*_ph,sat_ denotes the saturated photocurrent density at high *V*_eff_.

The effective internal voltage is given by:

$$\begin{aligned} V_{\mathrm{eff}}=V_{0}-V\#\left( S15 \right) \end{aligned}$$

Where *V*_0_ being the voltage at zero net current and *V* the applied voltage.^[7]^

**Note S9: Open-circuit Voltage (*V*_OC_) losses calculation**

The *V*_OC_ for a given bandgap is determined through detailed balance calculations following the Shockley-Queisser (S-Q) formalism. This analysis relies on nine fundamental assumptions:^[8]^

1. The incident photon flux corresponds to the ASTMG-173 standard AM 1.5G solar spectrum.

2. The absorber is a single-bandgap semiconductor with energy *E*_g_.

3. Perfect anti-reflection at the front surface (zero reflectivity).

4. Complete absorption of all photons with energy *E* > *E*_g._

5. No absorption of photons with energy *E* < *E*_g_.

6. Unity internal quantum efficiency (one electron-hole pair per absorbed photon).

7. Only radiative recombination is present.

8. Ideal reflection at the back surface.

9. Absence of parasitic losses from series or shunt resistances.

$$\begin{aligned} V_{OC}^{SQ}=0.932E_{g}-0.167 \#\left( S16 \right) \end{aligned}$$

This equation remains valid for both direct and indirect bandgap semiconductors within the spectral range of 0.9-2.2 eV. These limiting *V*_OC_ values can only be exceeded if one of the above assumptions becomes invalid (*e.g*. multi-exciton generation, hot-carrier collection, etc).

$$\begin{aligned} V_{OC}^{rad}=\frac{{QFLS}_{rad}}{q}\#\left( S17 \right) \end{aligned}$$

*QFLS*_rad_ is determined by a detailed balance calculation as equation S6.

Three components of Δ*V*_OC_ were obtained according to the balanced theory. The formulas are shown as follows:

$$\begin{aligned} \Delta V_{OC}=E_{g}{/q-V_{OC}=(E_{g}/q-V_{OC}^{SQ})-{(V}_{OC}^{SQ}-V}_{OC}^{rad})-{(V}_{OC}^{rad}-V_{OC}) \#\left( S18 \right) \end{aligned}$$

**Note S10: Fill factor (FF) losses calculation**

Under ideal conditions excluding non-radiative recombination and transport losses, the FF approaches the S-Q limit, which exhibits bandgap dependence. For the studied perovskite films with a bandgap of 1.68 eV, the S-Q limit was calculated as 90.91%. ^[8]^

When considering non-radiative recombination, but ignoring transport losses, the fill factor of a solar cell depends on factors such as temperature, ideal factor, and series and shunt resistances. Introducing a physical quantity *v*_OC_ for the normalized open-circuit voltage. The expression used for the calculation is:

$$\begin{aligned} v_{OC}=qV_{OC}/(nK_{B}T)\#\left( S19 \right) \end{aligned}$$

Where *V*_OC_ is open-circuit voltage, *K*_B_*T/q* is the thermal voltage and *n* is the WBG-PSCs ideality factor associated with the single-exponential model. The maximum FF (FF_max_) without charge transport losses can be empirically calculated using the following equation:

$$\begin{aligned} {FF}_{max}={[v}_{OC}-\ln\left( v_{OC}+0.72 \right)]/{(v}_{OC}+1)\#\left( S20 \right) \end{aligned}$$

For a more detailed analysis, transport losses can be further categorized into series parasitic resistance and shunt parasitic resistance. In this study, both types of resistance are considered as part of the overall transport losses and are not analyzed in depth.

**Note S11:** **series resistance (*R*_S_) and reverse saturation current (*J*_0_) calculation**

The series resistance (*R*_S_) can be obtained from the graph of −d*V/*d*J* versus (*J*_SC_−*J*)^−1^, and the reverse saturation current *J*_0_ can be obtained from the graph of ln (*J*_SC_−*J*) versus *V*+*R*_S_×*J*. These curves are derived from the current density-voltage (*J-V*) curves using the following equations:^[9]^

$$\begin{aligned} -\frac{dV}{dJ}=\frac{AK_{B}T}{q}{(J_{SC}-J)}^{-1}+R_{S}\#\left( S21 \right) \end{aligned}$$

$$\begin{aligned} ln(J_{SC}-J)=\frac{q}{AK_{B}T}(V+R_{S}\times J)+lnJ_{0}\#\left( S22 \right) \end{aligned}$$

Where *J* represents the current flowing through the external circuit, *J*_0_ is the reverse saturation current density, *A* is the ideality factor, *K*_B_ is the Boltzmann constant, *V* is the applied voltage, and *R*_S_ is the series resistance.

**Note S12: TPV and TPC characterizations**

Under small perturbation conditions, the TPV decay under varying bias light intensities (0.2, 0.4, 0.6, 0.8, and 1 sun) was fitted using a single-exponential decay function:

$$\begin{aligned} V=\Delta V_{0}\exp(-t/\tau_{\Delta V})\#\left( S23 \right) \end{aligned}$$

After fitting the TPV decays at different bias light intensities, the corresponding charge carrier lifetimes (*τ*_∆V_) and initial voltage amplitudes (*∆V*_0_) were obtained. The results reveal that *τ*_∆V_ exhibits an exponential dependence on the *V*_OC_ under the respective illumination conditions, as described by the following equation:

$$\begin{aligned} \tau_{\Delta V}=\tau_{{\Delta V}_{0}}\exp\left( -\beta V_{OC} \right) \#\left( S24 \right) \end{aligned}$$

After fitting, the values of the *τ*_∆V_ and the decay exponent (*β*) were determined. The corresponding charge variation (*∆Q*) was obtained by integrating the TPC decay. Subsequently, the capacitance (*C*) under varying light intensities was calculated using the following equation:

$$\begin{aligned} C=\Delta Q/\Delta V_{0}\#\left( S25 \right) \end{aligned}$$

The results demonstrate that the *C* exhibits an exponential dependence on the *V*_OC_ under the corresponding illumination intensity, as described by the following relationship:

$$\begin{aligned} C=C_{0}\exp(\gamma V_{OC})+D\#\left( S26 \right) \end{aligned}$$

After fitting, the baseline capacitance (*C₀*) and the exponential coefficient (*γ*) were determined. Subsequently, employing the following equation:

$$\begin{aligned} n=\frac{1}{Aed}\int_{-\infty}^{V\mathrm{oc}} C_{0}\exp(\gamma V_{OC})dV\#\left( S27 \right) \end{aligned}$$

Distinct values of the *n* were determined. Based on the preceding equation, equation (S21) was subsequently derived.^[10, 11]^

$$\begin{aligned} \lambda=\beta/\gamma\#\left( S28 \right) \end{aligned}$$

**Note S13: Calculation of Lead Sequestration Efficiency (SQE)**

The lead sequestration efficiency (SQE) was calculated based on the Pb^2+^ leakage rates from perovskite films and WBG-PSCs in deionized water (pH = 7). For perovskite films and WBG-PSCs, the SQE is calculated separately using the same formula:

$$\begin{aligned} \text{SQE}=\frac{R_{\text{control}}-R_{\text{target}}}{R_{\text{control}}}\times100\% \#\left( S29 \right) \end{aligned}$$

where *R*_control_ and *R*_target_ are the Pb^2+^ leakage rates of the control and target samples, respectively, for the specific type of sample (films or WBG-PSCs) under consideration.

**Supplementary Figures**

**Figure S1.** Optical images of CsPbI_3_ films without and with [SO_3_H-PMIm][OTf] perovskite films annealing at 90 °C.

**Figure S2.** The mechanism of reaction between [SO_3_H-PMIm][OTf] and CsI.

**Figure S3.** a) XPS spectra of I 3d. b) UV-vis absorption spectra of [SO_3_H-PMIm][OTf], PbI_2_ and PbI_2_ with [SO_3_H-PMIm][OTf] solution.

**Figure S4.** FTIR spectra of a,b) MAI and MAI + [SO_3_H-PMIm][OTf] and c,d) DMAI and DMAI + [SO_3_H-PMIm][OTf].

**Figure S5.** a) ^1^H NMR spectra of MAI, [SO_3_H-PMIm][OTf] and MAI + [SO_3_H-PMIm][OTf]. b,c) ^1^H NMR spectra of DMAI, [SO_3_H-PMIm][OTf] and DMAI + [SO_3_H-PMIm][OTf]. d) XPS spectra of N 1s.

**Figure S6.** a) PL and b) TRPL spectra of the control and target perovskite films. c) Computed differential lifetimes obtained by taking the derivative of b).

**Figure S7.** a) Schematic diagram of the principle of GIXRD measurement. b) Linear fit relationship of 2𝜃-sin^2^(ω).

**Figure S8.** Microstrain analysis of control and target perovskite films.

**Figure S9.** The longitudinal conductivity of the control and target perovskite films.

**Figure S10.** a) UV-vis spectra (inset: Tauc plot) and b) Urbach energy of the control and target perovskite films.

**Figure S11.** a) SCLC spectra of the electron-only devices. b) SCLC spectra of the hole-only device.

**Figure S12.** Statistic photovoltaic parameters of a batch of WBG-PSCs (100 WBG-PSCs in total, 20 WBG-PSCs for each group) fabricated with precursor solutions containing varying concentrations of [SO_3_H-PMIm][OTf] (0, 0.1, 0.2 and 0.3mg mL^-1^). a) *V*_OC_, b) *J*_SC_, c) FF, and d) PCE.

**Figure S13.** Nyquist plots of control and target WBG-PSCs.

**Fig. S14.** The *J*_SC_ values of WBG-PSCs of the control and target WBG-PSCs measured as a function of light intensity.

**Figure S15.** Dark *J-V* curves of the control and target WBG-PSCs.

**Figure S16.** a) The *V*_OC_ loss consists of radiative loss, perovskite loss (radiative loss below the bandgap), and interface loss of control and target WBG-PSCs. b) The FF S-Q limit and FF loss that contains charge-transport loss and non-radiative loss of the control and target WBG-PSCs.

**Figure S17.** a) Plots of -d*V*/d*J* versus (*J*_SC_-*J*)^-1^ with the linear fitting curves. b) Plots of ln (*J*_SC_-*J*) versus (*V*+*R*_S_×*J*) with the linear fitting curves.

**Figure S18.** TPV measurements were performed under different steady-state bias light intensities.

**Figure S19.** Photographs of the perovskite films dipped in water, corresponding to Figure 6d.

**Figure S20.** Parallel Pb^2+^ leakage tests on five independent pairs of a) perovskite films and b) corresponding devices during 120 min immersion in deionized water.

**Figure S21.** The operational photostability of unencapsulated WBG-PSCs under continuous 1-sun illumination in ambient air at room temperature according to ISOS-L-1. Error bars represent standard deviation (SD) from five independent samples (n = 5).

**Supplementary Tables**

**Table S1.** Double-exponential fitting parameters were obtained from the TRPL spectra.

| **Sample** | **τ_1_(ns)** | **τ_2_ (ns)** | **τ_ave_ (ns)** | **R^2^** |
| --- | --- | --- | --- | --- |
| **Control** | 8.93 | 60.65 | 9.65 | 0.993 |
| **Target** | 27.06 | 173.40 | 109.93 | 0.993 |

**Table S2.** The detailed photovoltaic parameters of the devices.

| **Device** | **Scan directions** | ***J*_SC_**  **(mA** **cm^-2^)** | | ***V*_OC_**  **(V)** | **FF**  **(%)** | **PCE**  **(%)** | **HI** |
| --- | --- | --- | --- | --- | --- | --- | --- |
| **Control** | Reverse | 21.40 | 1.2063 | | 79.34 | 20.48 | 0.066 |
| **Control** | Forward | 21.33 | 1.2048 | | 74.43 | 19.13 |  |
| **Target** | Reverse | 21.85 | 1.2314 | | 81.86 | 22.02 | 0.025 |
| **Target** | Forward | 21.95 | 1.2254 | | 79.81 | 21.47 |  |

Hysteresis index (HI): $\mathbf{HI=}\mathbf{(PCE}_{\mathbf{RS}}\boldsymbol{-}\mathbf{PCE}_{\mathbf{FS}}\boldsymbol{)/}\mathbf{PCE}_{\mathbf{RS}}$.

Table S3. Summary of pure-iodide wide-bandgap perovskite solar cells reported in the literatures.

| **Perovskite** | ***E*_g_ (eV)** | ***V*_OC_ (V)** | ***J*_SC_ (mA cm^-2^)** | **FF (%)** | **PCE (%)** | **Years** | **Ref.** |
| --- | --- | --- | --- | --- | --- | --- | --- |
| Cs_0.85_FA_0.15_PbI_3_ | 1.67 | 1.02 | 21.4 | 80.0 | 17.5 | 2020 | ^[12]^ |
| Cs_0.5_FA_0.5_PbI_3_ | 1.65 | 1.127 | 21.69 | 82.0 | 20.04 | 2021 | ^[13]^ |
| Cs_0.3_DMA_0.2_MA_0.5_PbI_3_ | 1.68 | 1.21 | 20.30 | 82.1 | 20.18 | 2022 | ^[14]^ |
| Cs_0.5_FA_0.5_PbI_3_ | 1.65 | 1.21 | 22.65 | 82.73 | 22.67 | 2023 | ^[15]^ |
| Cs_0.5_FA_0.5_PbI_3_ | 1.65 | 1.03 | 22.82 | 78.8 | 18.52 | 2023 | ^[16]^ |
| Cs_0.3_DMA_0.2_MA_0.5_PbI_3_ | 1.68 | 1.193 | 21.70 | 82.3 | 21.30 | 2023 | ^[17]^ |
| Cs_0.7_FA_0.3_PbI_3_ | 1.67 | 1.23 | 21.43 | 81.36 | 21.42 | 2025 | ^[18]^ |
| Cs_0.3_DMA_0.2_MA_0.5_PbI_3_ | 1.68 | 1.199 | 21.05 | 85.37 | 21.55 | 2025 | ^[19]^ |
| Cs_0.4_DMA_0.2_FA_0.2_MA_0.2_PbI_3_ | 1.67 | 1.204 | 21.80 | 83.19 | 21.84 | 2025 | ^[20]^ |
| Cs_0.3_DMA_0.2_MA_0.5_PbI_3_ | 1.68 | 1.196 | 20.80 | 82.00 | 20.40 | 2025 | ^[21]^ |
| Cs_0.504_FA_0.334_DMA_0.064_MA_0.098_PbI_3_ | 1.656 | 1.23 | 21.19 | 82.39 | 21.47 | 2025 | ^[22]^ |
| **Cs_0.3_DMA_0.2_MA_0.5_PbI_3_** | **1.68** | **1.231** | **21.85** | **81.86** | **22.02** | **2026** | **This work** |

**Table S4.** The fitted parameters from the EIS spectra.

| **Device** | ***R*_s_ (Ω)** | ***R*_ct_ (Ω)** | ***R*_rec_ (Ω)** |
| --- | --- | --- | --- |
| **Control** | 81.3 | 3.82 × 10^3^ | 3.20 × 10^5^ |
| **Target** | 81.1 | 2.87 × 10^3^ | 3.93 × 10^5^ |

**References**

[1]	C. Duan, F. Zou, S. Li, Q. Zhu, J. Li, H. Chen, Z. Zhang, C. Chen, H. Guo, J. Qiu, K. Wang, Y. Dong, Y. Qiu, L. Ding, X. Lu, H. Luo, K. Yan, *Adv. Energy Mater.* **2024**, *14*, 2303997.

[2]	Z. Zheng, F. Li, J. Gong, Y. Ma, J. Gu, X. Liu, S. Chen, M. Liu, *Adv. Mater.* **2022**, *34*, 2109879*.*

[3]	S. Zhang, F. Ye, X. Wang, R. Chen, H. Zhang, L. Zhan, X. Jiang, Y. Li, X. Ji, S. Liu, M. Yu, F. Yu, Y. Zhang, R. Wu, Z. Liu, Z. Ning, D. Neher, L. Han, Y. Lin, H. Tian, W. Chen, M. Stolterfoht, L. Zhang, W.-H. Zhu, Y. Wu, *Science* **2023**, *380*, 404-409.

[4]	J. Liu, M. De Bastiani, E. Aydin, G. T. Harrison, Y. Gao, R. R. Pradhan, M. K. Eswaran, M. Mandal, W. Yan, A. Seitkhan, M. Babics, A. S. Subbiah, E. Ugur, F. Xu, L. Xu, M. Wang, A. u. Rehman, A. Razzaq, J. Kang, R. Azmi, A. A. Said, F. H. Isikgor, T. G. Allen, D. Andrienko, U. Schwingenschlögl, F. Laquai, S. De Wolf, *Science* **2022**, *377*, 302-306.

[5]	S. Liu, J. Li, W. Xiao, R. Chen, Z. Sun, Y. Zhang, X. Lei, S. Hu, M. Kober-Czerny, J. Wang, F. Ren, Q. Zhou, H. Raza, Y. Gao, Y. Ji, S. Li, H. Li, L. Qiu, W. Huang, Y. Zhao, B. Xu, Z. Liu, H. J. Snaith, N.-G. Park, W. Chen, *Nature* **2024**, *632*, 536-542.

[6]	X. Zhang, W. Qiu, S. Apergi, S. Singh, P. Marchezi, W. Song, C. Sternemann, K. Elkhouly, D. Zhang, A. Aguirre, T. Merckx, A. Krishna, Y. Shi, A. Bracesco, C. van Helvoirt, F. Bens, V. Zardetto, J. D’Haen, A. Yu, G. Brocks, T. Aernouts, E. Moons, S. Tao, Y. Zhan, Y. Kuang, J. Poortmans, *ACS Energy Lett.* **2023**, *8*, 2532-2542.

[7]	C. Xu, P. Hang, C. Kan, X. Guo, X. Song, C. Xu, G. You, W. Q. Liao, H. Zhu, D. Wang, Q. Chen, Z. Hong, R. G. Xiong, X. Yu, L. Zuo, H. Chen, *Nat. Commun.* **2025**, *16*, 835.

[8]	C. Li, Y. Zhang, X. Zhang, P. Zhang, X. Yang, H. Chen, *Adv. Funct. Mater.* **2023**, *33*, 2214774.

[9]	H. Zhang, S. Yuan, Z. Qiu, Y. Jiang, X. Zhu, X. Wan, B. Cao, *J MATER SCI-MATER EL* **2018**, *29*, 8838-8846.

[10]	S. Wood, J. C. Blakesley, F. A. Castro, *Phys. Rev. Appl.* **2018**, *10*, 024038.

[11]	H. Yu, Y. Wang, X. Zou, J. Yin, X. Shi, Y. Li, H. Zhao, L. Wang, H. M. Ng, B. Zou, X. Lu, K. S. Wong, W. Ma, Z. Zhu, H. Yan, S. Chen, *Nat. Commun.* **2023**, *14*, 2323.

[12]	Z. Shao, H. Meng, X. Du, X. Sun, P. Lv, C. Gao, Y. Rao, C. Chen, Z. Li, X. Wang, G. Cui, S. Pang, *Adv. Mater.* **2020**, *32*, 2001054.

[13]	X. Wang, Y. Chen, T. Zhang, X. Wang, Y. Wang, M. Kan, Y. Miao, H. Chen, X. Liu, X. Wang, J. Shi, L. Zhang, Y. Zhao, *ACS Energy Lett.* **2021**, *6*, 2735-2741.

[14]	S. G. Ji, I. J. Park, H. Chang, J. H. Park, G. P. Hong, B. K. Choi, J. H. Jang, Y. J. Choi, H. W. Lim, Y. J. Ahn, S. J. Park, K. T. Nam, T. Hyeon, J. Park, D. H. Kim, J. Y. Kim, *Joule* **2022**, *6*, 2390-2405.

[15]	Y. Wei, Z. Shao, Z. Li, C. Gao, X. Sun, Q. Zhao, L. Hao, B. Zhang, D. Liu, X. Wang, C. Cui, Q. Huang, Y. Han, X. Wang, G. Cui, S. Pang, *ACS Energy Lett.* **2023**, *8*, 4726-4732.

[16]	R. Zhang, L. Li, W. Wang, Z. Wu, Y. Wang, J. Hong, H. Rao, Z. Pan, X. Zhong, *Adv. Funct. Mater.* **2023**, *33*, 2300552.

[17]	T. Nie, Z. Fang, T. Yang, K. Zhao, J. Ding, S. Liu, *Angew. Chem. Int. Ed.* **2024**, *63*, e202400205.

[18]	J. Kim, J. Park, G. Kim, W. Xu, S. D. Stranks, H. Min, S. I. Seok, *Small* **2025**, *21*, 2500197.

[19]	X. Zhou, X. Li, B. Shi, P. Wang, X. Du, Y. Zhao, X. Zhang, *ACS Nano* **2025**, *19*, 11187-11196.

[20]	D. Li, T. Nie, G. Zhao, R. Lv, J. Feng, J. Ding, S. Yang, S. Liu, Z. Fang, *Adv. Funct. Mater.* **2025**, *36*, e02847.

[21]	K. Wu, G. Du, X. Wang, C. Zhao, L. Yang, J. Wei, T. Li, P. Zhang, F. Hou, *J. Phys. Chem. Lett.* **2025**, *16*, 10785-10795.

[22]	G. P. Hong, B. Gil, S. J. Park, Y. J. Choi, Y. S. Yoon, M. Kim, J. Y. Kim, *Adv. Energy Mater.* **2025**, e05731.
